# Supplementary material for: Investigating the effect of relationship satisfaction on postpartum women’s health-related quality of life in Burkina Faso: a cross-sectional analysis
Source: BMJ Open. 2021 Sep 2;11(9):e048230. doi: 10.1136/bmjopen-2020-048230 (PMC8413953; doi:10.1136/bmjopen-2020-048230)
Supplement: Author's manuscript [file bmjopen-2020-048230.draft_revisions.pdf]

BMJ Open is committed to open peer review. As part of this commitment we make the peer review history of every article we publish publicly available.

When an article is published we post the peer reviewers' comments and the authors' responses online. We also post the versions of the paper that were used during peer review. These are the versions that the peer review comments apply to.

The versions of the paper that follow are the versions that were submitted during the peer review process. They are not the versions of record or the final published versions. They should not be cited or distributed as the published version of this manuscript.

BMJ Open is an open access journal and the full, final, typeset and author-corrected version of record of the manuscript is available on our site with no access controls, subscription charges or pay-per-view fees (http://bmjopen.bmj.com).

If you have any questions on BMJ Open's open peer review process please email info.bmjopen@bmj.com

BMJ Open

Investigating the effect of relationship satisfaction on postpartum women's health-related quality of life in Burkina Faso

Journal:	*BMJ Open*
Manuscript ID	bmjopen-2020-048230
Article Type:	Original research
Date Submitted by the Author:	21-Dec-2020

Complete List of Authors:	Lokubal, Paul; University of Oxford, Department of Population Health; London School of Hygiene & Tropical Medicine, Department of Population Health<br>Calvert, Clara; London School of Hygiene and Tropical Medicine, Department of Population Health<br>Cousens, Simon; London School of Hygiene & Tropical Medicine, Department of Infectious Disease Epidemiology<br>Daniele, Marina; King's College London, Women and Children's Health<br>Ganaba, Rasmané; CHU-SS Bobo Dioulasso<br>Filippi, Veronique; London School of Hygiene & Tropical Medicine, Department of Infectious Disease Epidemiology

Keywords:	PUBLIC HEALTH, HEALTH SERVICES ADMINISTRATION & MANAGEMENT, Health policy < HEALTH SERVICES ADMINISTRATION & MANAGEMENT

SCHOLARONE™
Manuscripts

I, the Submitting Author has the right to grant and does grant on behalf of all authors of the Work (as defined in the below author licence), an exclusive licence and/or a non-exclusive licence for contributions from authors who are: i) UK Crown employees; ii) where BMJ has agreed a CC-BY licence shall apply, and/or iii) in accordance with the terms applicable for US Federal Government officers or employees acting as part of their official duties; on a worldwide, perpetual, irrevocable, royalty-free basis to BMJ Publishing Group Ltd ("BMJ") its licensees and where the relevant Journal is co-owned by BMJ to the co-owners of the Journal, to publish the Work in this journal and any other BMJ products and to exploit all rights, as set out in our licence.

The Submitting Author accepts and understands that any supply made under these terms is made by BMJ to the Submitting Author unless you are acting as an employee on behalf of your employer or a postgraduate student of an affiliated institution which is paying any applicable article publishing charge ("APC") for Open Access articles. Where the Submitting Author wishes to make the Work available on an Open Access basis (and intends to pay the relevant APC), the terms of reuse of such Open Access shall be governed by a Creative Commons licence – details of these licences and which Creative Commons licence will apply to this Work are set out in our licence referred to above.

Other than as permitted in any relevant BMJ Author's Self Archiving Policies, I confirm this Work has not been accepted for publication elsewhere, is not being considered for publication elsewhere and does not duplicate material already published. I confirm all authors consent to publication of this Work and authorise the granting of this licence.

For peer review only

Investigating the effect of relationship satisfaction on postpartum women's health-related quality of life in Burkina Faso

Corresponding author: Lokubal Paul, PhD Student; National Perinatal Epidemiology Unit (NPEU), Nuffield Department of Population Health (NDPH), University of Oxford, Old Road Campus, Headington, Oxford OX3 7LF, paul.lokubal@ndph.ox.ac.uk / Department of Population Health, Faculty of Epidemiology and Population Health, The London School of Hygiene and Tropical Medicine, Keppel Street, Bloomsbury, London, WC1E 7HT, London, UK

Co-authors:

1. Clara Calvert, PhD; Department of Population Health, Faculty of Epidemiology and Population Health, The London School of Hygiene and Tropical Medicine, Keppel Street, Bloomsbury, London, WC1E 7HT, London, UK
2. Simon Cousens, MSc; Department of Infectious Disease Epidemiology, Faculty of Epidemiology and Population Health, The London School of Hygiene and Tropical Medicine, Keppel Street, Bloomsbury, London, WC1E 7HT, London, UK
3. Marina A.S. Daniele, PhD; Department of Women and Children's Health, School of Life Course Sciences, Faculty of Life Sciences and Medicine, King's College London, Becket House, London SE1 7EU, UK
4. Rasmané Ganaba, PhD; AfricSante, Bobo-Dioulasso, Burkina Faso
5. Veronique Filippi, PhD; Department of Infectious Disease Epidemiology, Faculty of Epidemiology and Population Health, The London School of Hygiene and Tropical Medicine, Keppel Street, Bloomsbury, London, WC1E 7HT, London, UK

Word count: 3337

Key words: quality of life; relationship satisfaction; postpartum; Burkina Faso; male partners

For peer review only - http://bmjopen.bmj.com/site/about/guidelines.xhtml

ABSTRACT

Introduction: The period following childbirth poses physiological, physical, social, and psychological challenges to women that may affect their quality of life. Few studies in Africa have explored women's health-related quality of life (HrQoL) and its determinants in postpartum populations, including the quality of women's relationships with their male partners. We investigated whether relationship satisfaction was associated with better HrQoL among postpartum women in Burkina Faso, 8 months after childbirth.

Methods: We analysed data from 547 women from the control arm of a randomized controlled trial in Burkina Faso. The study outcome was a woman's HrQoL, assessed using the cross-culturally validated World Health Organisation QOL-BREF tool, with response categories adapted for Burkina Faso. The exposure was relationship satisfaction measured using questions adapted from the Dyadic Adjustment Scale and Marital Assessment Test tools. We calculated the median HrQOL scores for the study sample, overall and for each domain of HrQOL (physical, psychological, social and environmental). The association between relationship satisfaction and HrQoL was examined using multiple linear regression models with robust standard errors.

Results: Postpartum women had high median HrQoL scores in the physical (88.1), psychological (93.1), social (86.1) and environmental (74.0) domains and overall HrQoL (84.0). After adjusting for confounders, we observed strong evidence for an association between relationship satisfaction and the HrQoL for the physical (p-value for linear test for trend=0.010), social (p = 0.001), and environmental (p-value<0.001) domains and overall HrQoL (p-value<0.001) and weak evidence of an association with the psychological domain (p = 0.070). We find that higher relationship satisfaction is associated with increased HrQoL, overall and across all the domains.

For peer review only - http://bmjopen.bmj.com/site/about/guidelines.xhtml

Conclusion: Higher relationship satisfaction is associated with higher HrQoL scores. Policies should aim to increase male involvement in supporting their partners to cope with the challenges of childbirth and childcare in the postpartum period.

Strengths and Limitations

- This is one of the few studies to explore HrQoL during the extended postpartum period in an African setting, whereas most studies have documented it during pregnancy or the immediate postpartum period.
- Data collection on HrQoL and relationship satisfaction took place at one point in time eight months after childbirth and we cannot rule out reverse causality in which women who have high HrQoL scores are consequently more likely to feel satisfied with their relationship
- We did not validate the tools locally for collecting the outcomes and the main exposure although we adapted them to suit the local context and also conducted rigorous pretesting to ensure it was adequately understood by the participants

For peer review only - http://bmjopen.bmj.com/site/about/guidelines.xhtml

INTRODUCTION

The 1948 definition of health by the World Health Organisation (WHO) as "*a state of complete physical, mental and social well-being and not merely the absence of disease or infirmity*",[1] revolutionised the concept of health and led to major advances in its assessment beyond traditional health status indicators of morbidity and mortality. This definition led to the development of subjective concepts such as quality of life (QoL),[2]. According to WHO, QoL is defined as *"individuals' perceptions of their position in life in the context of the culture and value systems in which they live and in relation to their goals, expectations, standards and concerns*",[2, 3]. It is a multidimensional concept that captures all spheres of a person's life; it includes physical, mental, social and functional aspects of an individual's life and their satisfaction with it,[4]. According to Torrance, health-related quality of life (HrQoL) is "*a subset [of QoL] relating only to the health domain of that [person's] existence*",[5].

Following childbirth, many women experience physical, emotional, functional, social and psychological challenges that can affect their wellbeing,[6–10]. For example, one study of women 9-12 months postpartum in the US found that to 69% of women experienced at least one physical symptom following childbirth,[7], while another study conducted In Spain reported that 85% of women experienced fatigue six weeks after childbirth,[8]. Some of these challenges are known to persist well beyond the traditional six week postpartum period and are associated with poor overall HrQoL among postpartum women,[7, 11].

For peer review only - http://bmjopen.bmj.com/site/about/guidelines.xhtml

The reasons for poor health-related quality of life noted amongst women in the postpartum period are complex and are likely to be driven by a range of different factors including, for example, clinical experiences (i.e. if any complications occurred during delivery), and socio-demographic and economics characteristics,[12,19,23–25]. There is a growing body of evidence pointing to the important role that relationship quality plays in HrQoL for women, including during pregnancy and in the postpartum period,[16–20]. There are many possible mechanisms through which higher relationship quality may lead to improved HrQoL among postpartum women including existence of emotional connection between spouses which helps to protect against postpartum depression,[16, 21]; easy resolution of conflicts and sharing of childcare responsibilities,[12], and social, financial and emotional support following childbirth,[22, 23]. There are only a few, generally small, studies that have directly examined the influence of relationship satisfaction on women's postpartum HrQoL. In these studies, spousal support,[22, 23], staying with the partner postpartum,[24] and women's marital satisfaction with their spouses,[16–20] are associated with higher HrQoL among postpartum women.

Improving maternal wellbeing is a key priority with the Sustainable Development Goals agenda,[25]. Given the paucity of studies conducted in Sub-Saharan African on women's postpartum HrQoL and the interest in the impact of male partner involvement on women's health and wellbeing,[26], we analysed data from 547 women who were enrolled in the control arm of a randomised controlled trial to investigate the effects of relationship satisfaction on postpartum women's HrQoL in Burkina Faso.

For peer review only - http://bmjopen.bmj.com/site/about/guidelines.xhtml

METHODS

Dataset and study design

The study is a secondary analysis of data from the control arm of an individual, non-blinded randomised-controlled trial (RCT) conducted in five primary health care facilities in three health districts in the city of Bobo-Dioulasso, Burkina Faso. The data were collected between 16th February 2015 and 4th July 2016. The details of the study design, data collection methods and data management, and ethical approval are described in detail elsewhere,[26]. In brief, the trial investigated the effectiveness of a health services intervention designed to increase male partners' involvement in maternity care, hypothesising that the intervention would increase postnatal care attendance, the duration of exclusive breastfeeding and the use of postpartum contraception[26]. Women were eligible for the trial if they were aged 15-45 years and living with a male partner,[26]. A total of 1114 women (583 in the intervention arm and 561 in the control group) were enrolled in the study and followed from around 20 weeks of gestation up to eight months postpartum,[26].

Ethics

This was a secondary data analysis of a clinical trial registered with ClinicalTrials.gov (NCT02309489). The ethics for this project was obtained from LSHTM MSc Ethics and Research Committee (Reference Number 16964).

For peer review only - http://bmjopen.bmj.com/site/about/guidelines.xhtml

Patient and public involvement

This was a secondary data analysis of a primary study and while full participant consent was obtained in the primary study, it was not required for this analysis and publication.

Variables

Outcome: health-related quality of life

The outcome for this analysis is HrQoL and its four domains, measured at eight months postpartum [3]. Data on HrQoL were collected using the French version of the World Health Organisation QOL-BREF (WHOQOL-BREF) tool,[2]. This tool has been validated for use in general population in Africa, but not specifically Burkina Faso, so response options were adapted to the local context by the study authors to reflect the low formal education level among women and to address difficulties in differentiating between response options in local languages,[26]. The WHOQOL-BREF is a 26-item quality of life assessment tool covering four domains: physical (7 items – enquired about pain and discomfort; energy and fatigue; sleep and rest; dependence on medication; mobility; activities of daily living and working capacity), psychological (six items – enquired about positive feelings; negative feelings; self-esteem; thinking learning; memory and concentration; body image; spirituality, religion and personal beliefs), social (three items – personal relations; sex; and practical social support), and environmental (eight items – financial resources; information and skills; recreation and leisure; home environment; access to health and social care; physical safety and security; physical environment; and transport). There are two further questions on general quality of life and general health,[4]. The items probed women's perceptions

For peer review only - http://bmjopen.bmj.com/site/about/guidelines.xhtml

about their life in the four weeks preceding the questionnaire. For this study, each item was rated on a 3-point Likert Scale (i.e., dissatisfied/low=1, neither satisfied nor dissatisfied/moderate/average=2, satisfied/very much/high=3) with lower scores indicating lower HrQoL and the higher scores higher HrQoL. We summed these scores to give an overall HrQoL score and individual domain scores for each individual. We then transformed the respective scores to a 20-point scale and subsequently to a 100-point score scale as per WHO instructions,[3] and analysed it as a continuous variable.

Exposure: relationship satisfaction

The primary predictor for this study was relationship satisfaction measured at eight months postpartum. We defined "relationship satisfaction" in the postpartum period as a "*woman's satisfaction with her relationship with her partner and the degree of communication, shared decision-making and agreement between the couple,*[26]. We calculated the relationship satisfaction score using a combination of 35 pretested questions adapted from the Marital Adjustment Test and Dyadic Adjustment Scale,[27, 28] but not locally validated for use in Burkina Faso. Women were asked about their satisfaction with their partner, satisfaction with degree of communication, satisfaction with shared decision-making and agreement on 12 questions (family finances, the woman's own family, the woman's in-laws, number of children, children's health, children's education, children's nutrition, man's work, woman's work, time the couple spent together, family planning and co-spouses or other women). Lower scores on these questions indicate lower satisfaction. We summed the scores from each individual question across all the questions to generate the total score for each. We then divided

For peer review only - http://bmjopen.bmj.com/site/about/guidelines.xhtml

For peer review only

relationship satisfaction score into the following equal categories: very low, low, moderate, and high.

Potential confounders

We considered the following variables as potential confounders of the association between relationship satisfaction and HrQoL: women's age at enrolment (15-24, 25-34 and 35+); women's education level (none, primary, and secondary or higher); male partner's age at enrolment (20-29, 30-39 and 40+); male partner's education level (none, primary, secondary, and tertiary); type of marriage (polygamy and monogamy); woman's postpartum place of residence (with her parents/in-laws and with male partner); source of finance for women's health care after childbirth (divided into four categories of did not attended care, paid for herself, care was paid for by partner, and care was paid for by others); woman's need for contraception (not met and met); and woman's views on domestic violence (justified for at least one reason and not justified at all).

Statistical analysis

We examined the distribution of participants' characteristics at baseline and at eight months using percentages for categorical variables and means, standard deviations, medians and interquartile ranges for continuous variables.

We examined the internal consistency and reliability of both the WHOQOL-BREF tool and relationship satisfaction index using Cronbach's alpha coefficient, which assesses how well a set of items, variables or attributes measures a single, one-dimensional latent construct among participants involved in a study. We considered Cronbach's

For peer review only - http://bmjopen.bmj.com/site/about/guidelines.xhtml

alpha coefficient values 0.70 and above satisfactory,[29]. We then calculate the median and interquartile ranges for the HrQoL (overall score and for each domain). This was done for the total sample of women and also stratified by relationship satisfaction.

In the multivariate analysis, we considered all the other variables potential confounders for the association between relationship satisfaction and HrQoL. Due to presence of heteroskedasticity, we ran a multiple linear regression models with robust standard errors to estimate adjusted regression coefficients, adding all the variables to the model simultaneously. We analysed the data using STATA/SE 15.1.

RESULTS

Of the 561 women enrolled in the control arm of the study, 14 were missing at the eight months' postpartum follow-up period: 11 were lost-to-follow-up without further information, one moved away, one withdrew voluntarily from the study and one died. Thus, the analysis is based on 547 women who were still enrolled in the study at eight months postpartum.

Table 1 shows women's and their partner's characteristics at baseline and at 8 months. The relationship satisfaction score ranged from 3 to 56, with a mean score of 30.5 (standard deviation (SD) = 10.1), median score of 30 and interquartile range (IQR) of 13 (24-37). The Cronbach's alpha coefficient for relationship satisfaction was 0.849. The

For peer review only - http://bmjopen.bmj.com/site/about/guidelines.xhtml

youngest woman was 16 years and the oldest was 43 years old. The median age of the women was 26 years with an IQR of 9 years (21-30 years) and only 64 (12%) were 35 years and older. About 50% of women had no formal education and 85% were in a monogamous relationship. There was information available on 492 male partners with a median age of 33 years (IQR 29-39 years). The youngest was 20 years and the oldest was 62 years old and about half (49%) of the men were aged 30-39 years. Over 70% (358) of the men had either no education or had attained primary education only. Eighty-five percent (457) of women stayed at the partner's place postpartum, 64% (346) had their health care paid for by their partners while 81% (438) had met need for family planning while 61% did not view domestic violence justifiable at all.

Table 1: Demographic Characteristics of Women at the Baseline and 8 Months (number, N=547)

Variable	Number (n)	Percent (%)
**Relationship Satisfaction***		
Very Low (0-23)	131	24.6
Low (24-29)	117	22.0
High (30-36)	135	25.3
Very High (37-56)	150	28.1
**Age (in years)**		
15-24	233	42.6
25-34	250	45.7
35+	64	11.7
**Education level reached**		
None	271	49.5
Primary	163	29.8
Secondary or higher	113	20.7
**Type of Marriage**		
Polygamous	84	15.4
Monogamous	463	84.6
**Men's age (in years) ****		
20-29	133	27.0
30-39	240	48.8
40+	119	24.2

For peer review only - http://bmjopen.bmj.com/site/about/guidelines.xhtml

**Male partner's education level reached*** **		
None	236	47.9
Primary	122	24.8
Secondary	87	17.7
Tertiary	48	9.7
**Woman's Postpartum Place of Residence ******		
In-laws/own Parents	82	15.2
Husband	457	84.8
**Funding for woman's care******		
Did not attend care	91	16.9
Woman	63	11.7
Partner	346	64.2
Others	39	7.2
**Meet need for family planning******		
Unmet	101	18.7
Met	438	81.3
**Views on domestic violence******		
Justified for at least one reason	210	39.0
Not justified at all	329	61.0

** Data on only 533 observations available ** Data on only 492 observations available*
**** Data on only 493 observations available **** Data on only 539 observations available*

Error! Reference source not found. shows transformed HrQoL scores with medians and interquartile ranges (IQR). Overall, we observed excellent internal consistency across all 26 questions with a Cronbach's alpha coefficient of 0.853. For the individual domains Cronbach's alpha was as follows:0.703 for physical, 0.721 for psychological, 0.383 for social and 0.555 for environmental. In this population, the median overall HrQoL score was 84.0 (IQR 14.4).

Table 2: Transformed HrQoL Scores with Cronbach's Alpha Coefficient (N=547)

Domains	Number	Mean	Standard deviation	Median	25th percentile	75th percentile	Cronbach's alpha
**Physical***	546	85.0	12.9	88.1	82.1	94.0	0.703
**Psychological****	545	88.4	13.4	93.1	79.2	100	0.721
**Social****	545	88.0	14.0	86.1	86.1	100	0.383
**Environmental****	545	74.5	12.9	74.0	63.5	84.4	0.550
**Overall*****	540	83.0	10.5	84.0	76.0	90.4	0.853

1 observation missing; **2 observations missing; * 7 observations missing*

For peer review only - http://bmjopen.bmj.com/site/about/guidelines.xhtml

Table shows the mean scores and mean regression coefficients with robust standard errors of the association between HrQoL and its domains with relationship satisfaction. We observed higher mean scores in the psychological and social domains while the environmental domain had the lowest mean scores across all the four relationship satisfaction categories. After adjusting for all potential confounders, we found a dose-dependent relationship between relationship satisfaction and the HrQoL for the physical (p-value for linear test for trend=0.010), social (p =0.001), and environmental (p < 0.001) domains and overall HrQoL (p < 0.001). We found weak evidence for an association of linear trend between relationship satisfaction and the psychological domain of HrQoL (p = 0.070). We observed that there is a trend across most of the domains and overall HrQoL with higher relationship satisfaction being associated with higher HrQoL scores. We also observed no differences between high and very low relationship satisfaction in the psychological domain, and between moderate and very low relationship satisfaction in the social domain. In all domains and overall HrQoL, we found no differences between in HrQoL very low and low relationship satisfaction.

*Table 3: Association between Relationship Adjustment and HrQoL Domains using multiple quantile regression with robust standard errors**

**HrQoL**	**Unadjusted mean scores**			**Adjusted regression coefficients**			
**Relationship Satisfaction**	Mean	SD	p-value	Coef.	95% CI		p-value[1]
**Physical**							
Very Low	83.1	13.3	0.026	0.00			0.010
Low	83.6	13.1		0.85	-2.83	4.53	
Moderate	86.6	12.6		3.92	0.45	7.39	
High	86.7	11.8		4.15	0.46	7.83	
**Psychological**							
Very Low	86.2	16.6	0.040	0.00			0.070
Low	88.6	13.2		2.41	-1.12	5.94	
Moderate	90.5	13.4		3.51	-0.04	7.05	
High	89.7	11.0		3.41	-0.46	7.28	
**Social**							

For peer review only

For peer review only - http://bmjopen.bmj.com/site/about/guidelines.xhtml

Very Low	84.3	16.6	<0.001	0.00			0.001
Low	87.1	12.9		2.23	-1.74	6.20	
Moderate	88.1	13.5		2.30	-1.45	6.04	
High	92.2	11.1		6.34	2.80	9.87	
**Environmental**							
Very Low	70.9	13.4	0.001	0.00			<0.001
Low	73.6	11.8		2.43	-1.10	5.96	
Moderate	76.0	12.2		4.16	0.83	7.50	
High	77.7	12.8		6.79	3.46	10.12	
**Overall**							
Very Low	80.3	11.4	0.001	0.00			<0.001
Low	82.0	9.7		1.52	-1.22	4.26	
Moderate	84.4	10.1		3.54	0.84	6.24	
High	85.5	9.8		4.97	2.20	7.73	

**Adjusted for age, education level, type of marriage, male partner's age, male partner's education level, woman's postpartum place of residence, funding for woman's health care and met need for contraception. SD = Standard Deviation; CI = Confidence Interval; [1]=linear test for trend p-value*

DISCUSSION

Key Findings

Overall, this study shows that higher relationship satisfaction scores are associated with better HrQoL among postpartum women in Burkina Faso for all the individual domains of HrQoL except for the psychological domain for which we found only weak evidence of an association with relationship satisfaction.

The dose-response association between relationship satisfaction and the overall HrQoL and the physical, social and environmental domains is consistent with findings from studies elsewhere that demonstrate that higher marital or relationship satisfaction is associated with an improved HrQoL among postpartum women,[11, 17, 30–33]. Higher relationship satisfaction is likely to be related to women's assessments of better spousal social, economic and emotional support in the postpartum period, factors known to be associated with higher HrQoL overall,[22, 24, 34–36]. Better spousal support has been

For peer review only - http://bmjopen.bmj.com/site/about/guidelines.xhtml

shown to lead to reductions in stress, anxiety during pregnancy, childbirth and in the postpartum period, offsetting some of the challenges of coping with parenthood and child care,[24]. In our study, we defined relationship satisfaction in relation to shared decision making, agreement on key family issues and communication between the couples. Couples who frequently discuss, agree, and make joint decisions on key family issues are likely to support each other to achieve their desired targets and thus have a higher HrQoL than those who do not. Increased spousal support may be responsible for the observed association between better relationship scores with HrQoL. However, women in Burkina Faso typically receive support from their extended family and social network, often in the form of financial and other assistance (such as helping in the care of the newborn) following childbirth,[9]. Thus, factors other than spousal support may explain the high HrQoL reported by women in our study.

In our study, we only found weak evidence for differences in the psychological domain of HrQoL between the four categories of relationship satisfaction. These findings are somewhat at odds with most of the literature regarding relationship satisfaction and HrQoL. For example, Whisman et al., showed that relationship adjustment or satisfaction predicted psychological symptoms such as anxiety and depression during both pregnancy and postpartum,[17] while another study exploring the relationship between quality of life with marital satisfaction among 103 Iranian Nurses in a hospital setting showed that marital satisfaction was associated with psychological wellbeing,[12]. A possible explanation for this weak association in our study is that relationship satisfaction in this community is essentially a social, physical and material (environmental) concept and about the ability of men to provide for their partners in the

For peer review only - http://bmjopen.bmj.com/site/about/guidelines.xhtml

postnatal period (social and environmental domains), in a setting where the health system is weak and poverty widespread. This hypothesis is consistent with findings from a Malawian study in which the quality of a relationship is conceptualised in terms of intimacy, partner support, sexual satisfaction, gender roles, decision-making, and communication and conflict management but not in terms of psychological wellbeing,[37].

Our results also show that overall, women had high HrQoL scores at eight months postpartum. In a study a validating the WHOQOL-BREF tool among Australian postpartum women with postnatal depression (PND) and those without PND, women without PND had higher mean scores compared to those PND; mean overall score for HrQoL for women without PND was 15.8 in the 20-score scale (equivalent to 73.9 in the 100-score scale) and the highest mean score of HrQoL for women without PND was observed in the environmental domain (16.2 in the 20-score scale, equivalent to 76.3 when transformed to the 100-score scale) while the lowest was observed in the psychological scale (15.5/20 or 71.9/100),[38]. In our study, however, the highest mean score was observed in the psychological domain with the lowest obtained in the environmental domain. The differences are likely due to sociodemographic and economic differences between Australian women (high-income country) and those from our study (low-income country). Whereas most studies show that women's postpartum HrQoL decreases following childbirth, this period also often brings joy to women and their partners and may be associated with improved HrQoL,[39]. It is also possible that any childbirth related-health issues, for example postpartum depression, will have resolved by eight months and women may have adapted to the demands of caring for a

For peer review only - http://bmjopen.bmj.com/site/about/guidelines.xhtml

new infant. Other studies suggest that HrQoL is related to time from childbirth. A longitudinal study comparing perinatal and postnatal HrQoL among 465 women in semi-rural Bangladesh showed that overall mean HrQoL scores increased by 37% from the perinatal period (within 2 to 7 days) to the postpartum period (within 40-60 days),[40]. The same study also showed that whereas 58% of women experienced moderate or severe levels of health problems in the perinatal period, these (health problems) resolved in the postpartum period and women experienced a high quality of life,[40]. Another prospective longitudinal study of Iranian women at 6-8 and 12-14 weeks postpartum showed that women had higher HrQoL scores in the 12-14 weeks' period compared to 6-8 weeks,[41]. In contrast, a study comparing HrQoL among Nigerian women at 6, 12 and 18 weeks postpartum showed that employed women, with only three months' maternity leave, had the highest scores at 12 weeks and the lowest scores at 18 weeks,[42]. However, the duration from childbirth differs markedly between women in our study (8 months) from most of the women studied.

Study Strengths and Limitations

This is one of the few studies to explore HrQoL during the extended postpartum period in an African setting, whereas most studies have documented it during pregnancy or the immediate postpartum period.

Our study has several limitations. It was conducted in Bobo-Dioulasso, an urban setting where access to health care is relatively easy and does not therefore represent the experience of women in the entire country. Data collection on HrQoL and relationship satisfaction took place at one point in time eight months after childbirth and we cannot

For peer review only - http://bmjopen.bmj.com/site/about/guidelines.xhtml

rule out reverse causality in which women who have high HrQoL scores are consequently more likely to feel satisfied with their relationship. In addition, we did not validate the tools locally for collecting the outcomes and the main exposure although we adapted them to suit the local context and also conducted rigorous pretesting to ensure it was adequately understood by the participants. However, both tools had good internal reliability consistency (relationship satisfaction 0.840 and HrQoL 0.853). Although we did not compare the 5-rated and 3-rated Likert scales, the good internal consistency of the 3-rated Likert scale suggests that it may be preferable in situations where many variables need to be collected in a short time with resources are limited and participants have low education levels which may present semantic challenges that make it difficult to distinguish between some response options in the questionnaire. However, a validation of the WHOQOL-BREF tool for use among postpartum women in SSA is needed.

Our study may have also suffered from several response biases such as social desirability bias, satisficing, acquiescence, and positive biases. Finally, we did not include in the analysis all potential confounders of the association between relationship satisfaction and HrQoL such as sex of the babies, delivery mode and employment that are known to affect HRQOL among postpartum women. This was due to the small number of observations in some categories, the long-time lag between the occurrence of an event (such as type of delivery) and the administration of the questions, and most of our study participants did not have meaningful gainful employment and as such, categorisation was difficult.

For peer review only - http://bmjopen.bmj.com/site/about/guidelines.xhtml

CONCLUSION

These findings enhance our understanding of the association between relationship satisfaction and health-related quality of life among postpartum women in general, but particularly among women in Sub-Saharan Africa. Policies that support male involvement in joint decision-making and support of their partners in the postpartum period need to be considered by sexual and reproductive health policy makers. More research into the impact of relationship satisfaction and other determinants including family social support on HrQoL among postpartum women over the entire postpartum period, from early to late postpartum period, is needed to ascertain how HrQoL may vary with time and how health services can best address maternal wellbeing and its determinants.

FUNDING

This study was funded by the Strengthening Evidence for Programming on Unintended Pregnancy (STEP UP) Research Program Consortium (grant code EPIDHC20) of which Veronique Filippi is a member, and by a contribution from the Faculty of Epidemiology and Population Health, London School of Hygiene and Tropical Medicine. STEP UP is funded by UKAid from the Department for International Development.

ACKNOWLEDGEMENTS

The authors wish to acknowledge the Economic and Social Research Council (ESRC) which funded Marina Daniele's studies at the London School of Hygiene and Tropical

For peer review only - http://bmjopen.bmj.com/site/about/guidelines.xhtml

Medicine and the Chevening Scholarship and the London School of Hygiene and Tropical Medicine which funded Paul Lokubal's MSc studies at the London School of Hygiene and Tropical Medicine.

CONTRIBUTORS

MD and VF conceived the idea, planned and designed the primary study. LP wrote the first draft while SC reviewed and provided statistical input. CC, SC, MD, RG and VF edited the draft and provided critical insights. All authors have approved and contributed to the final submitted manuscript.

DATA AVAILABILITY STATEMENT

All data relevant to the analysis are uploaded as supplementary information and is with the editors. Re-use is permitted given the authors are acknowledged in any publication. Further data may be obtained by emailing MD.

COMPETING INTERESTS

The authors declare no competing interests; no support from any organisation for the submitted work; no financial relationships with any organisations that might have an interest in the submitted work in the previous 3 years; no other relationships or activities that could appear to have influenced the submitted work.

REFERENCES

[1] World Health Organization (WHO). *Constitution of the World Health Organization*. 1948.

For peer review only - http://bmjopen.bmj.com/site/about/guidelines.xhtml

[2] Skevington SM, Lotfy M, O'connell KA. *The World Health Organization's WHOQOL-BREF quality of life assessment: Psychometric properties and results of the international field trial. A Report from the WHOQOL Group*. 2004.

[3] World Health Organization (WHO). Introduction, Adminstration, Scoring and Generic Version of the Assessment: WHOQOL-BREF.

[4] World Health Organization (WHO). WHOQOL: Measuring Quality of Life.

[5] Torrance GW. Utility approach to measuring health-related quality of life. *J Chronic Dis* 1987; 40: 593–603.

[6] Collaborators G 2013 Daly and H. Global, regional, and national disability-adjusted life years (DALYs) for 306 diseases and injuries and healthy life expectancy (HALE) for 188 countries, 1990-2013: quantifying the epidemiological transition GBD 2013 DALYs and HALE Collaborators *. *Lancet (London, England)*. Epub ahead of print 2015. DOI: 10.1016/S0140-6736(15)61340-X.

[7] Webb DA, Bloch JR, Coyne JC, et al. Postpartum physical symptoms in new mothers: their relationship to functional limitations and emotional well-being. *Birth* 2008; 35: 179–87.

[8] Miguel Martínez-Galiano J, Hernández-Martínez A, Rodríguez-Almagro J, et al. Women's Quality of Life at 6 Weeks Postpartum: Influence of the Discomfort Present in the Puerperium. Epub ahead of print 2019. DOI: 10.3390/ijerph16020253.

[9] Storeng KT, Drabo S, Filippi V. Too poor to live? A case study of vulnerability and maternal mortality in Burkina Faso. *Glob Health Promot* 2013; 20: 33–38.

[10] Filippi V, Ganaba R, Calvert C, et al. After surgery: The effects of life-saving caesarean sections in Burkina Faso. *BMC Pregnancy Childbirth*; 15. Epub ahead of print 2015. DOI: 10.1186/s12884-015-0778-7.

[11] Saurel-cubizolles M, Romito, Lelong N, et al. Women ' s health after childbirth : a longitudinal study in. 2000; 107: 1202–1209.

[12] Gharibi M, Sanagouymoharer G, Yaghoubinia F. The Relationship between Quality of Life with Marital Satisfaction in Nurses in Social Security Hospital in Zahedan. *Glob J*

For peer review only - http://bmjopen.bmj.com/site/about/guidelines.xhtml

Health Sci; 8. Epub ahead of print 2016. DOI: 10.5539/gjhs.v8n2p178.

[13] Nussbeck F, Bodenmann G. Long-Term Prediction of Relationship Satisfaction and Stability by Stress , Coping , Communication , and Well-Being. Epub ahead of print 2014. DOI: 10.1080/10502556.2014.931767.

[14] Basharpoor S, Sheykholeslami A. The relation of marital adjustment and family functions with quality of life in women. *Eur J Psychol* 2015; 11: 432–441.

[15] Margelisch K, Schneewind KA, Violette J, et al. Marital stability, satisfaction and well-being in old age: variability and continuity in long-term continuously married older persons. *Aging Ment Heal* 2017; 21: 389–398.

[16] Mobarakabadi A, Fallahchai R, Askari M. The Relationship between Marital Satisfaction and Postpartum Depression in Women who Visited Health Centers in Bandar Abbas city. *J Appl Environ Biol Sci* 2014; 4: 120–124.

[17] Whisman MA, Davila J, Goodman SH. Relationship Adjustment, Depression, and Anxiety During Pregnancy and the Postpartum Period. Epub ahead of print 2011. DOI: 10.1037/a0023790.

[18] Tendais I, Figueiredo B, Canário C, et al. Couples' psychological adjustment to twin parenthood: mode of conception (spontaneous versus assisted reproduction) and gender differences. Epub ahead of print 2019. DOI: 10.1017/S1463423618000269.

[19] Ifeoma Odinka J, Nwoke M, Chukwuorji JC, et al. Dependent Personality, Mindful Awareness, and Marital Satisfaction and their Association with Postpartum Psychological Distress in Two Tertiary Hospitals, Southeast Nigeria. *J Health Care Poor Underserved* 2020; 31: 920–938.

[20] Gameiro S, Nazaré B, Fonseca A, et al. Changes in marital congruence and quality of life across the transition to parenthood in couples who conceived spontaneously or with assisted reproductive technologies. *Fertil Steril* 2011; 96: 1457–1462.

[21] Odinka JI, Nwoke M, Chukwuorji JC, et al. Post-partum depression, anxiety and marital satisfaction: A perspective from Southeastern Nigeria. *South African J Psychiatry* 2018; 24: 1–8.

For peer review only - http://bmjopen.bmj.com/site/about/guidelines.xhtml

[22] Akbay AS, Taşçı-Duran E. How does spousal support affect women's quality of life in the postpartum period in Turkish culture? *Asian Women* 2018; 34: 29–45.

[23] Storeng KT, Akoum MS, Murray SF. This year i will not put her to work: The production/reproduction nexus in Burkina Faso. *Anthropol Med* 2013; 20: 85–97.

[24] Fontenele De Oliveira M, Parker L, Ahn H, et al. Maternal Predictors for Quality of Life during the Postpartum in Bra-zilian Mothers. *Health (Irvine Calif)* 2015; 7: 371–380.

[25] World Health Organization. Interventions for Improving Maternal and Newborn Health. *World Health*.

[26] Daniele MA, Ganaba R, Sarrassat S, et al. Involving male partners in maternity care in Burkina Faso: a randomized controlled trial. *Bull World Heal Organ* 2018; 96: 450–461.

[27] Locke HJ, Wallace KM, Funk JL&, et al. *Marital Adjustment Test (MAT) Reference*. 2007.

[28] Spanier GB. Dyadic adjustment scale. *Journal of Sex and Marital Therapy*. Epub ahead of print 1979. DOI: 10.1080/00926237908403730.

[29] Taber KS. The Use of Cronbach's Alpha When Developing and Reporting Research Instruments in Science Education. *Res Sci Educ* 2018; 48: 1273–1296.

[30] Woolhouse H, McDonald E, Brown S. Women's experiences of sex and intimacy after childbirth: Making the adjustment to motherhood. *J Psychosom Obstet Gynecol* 2012; 33: 185–190.

[31] Marie Dyrdal G, Røysamb E, Bang Nes R, et al. Can a Happy Relationship Predict a Happy Life? A Population-Based Study of Maternal Well-Being During the Life Transition of Pregnancy, Infancy, and Toddlerhood. Epub ahead of print 2010. DOI: 10.1007/s10902-010-9238-2.

[32] Midmer DK. *Family of origin, marital adjustment and the birth of the first child*. 1997.

[33] Luppi F, Carlo Alberto C. Adjustment to parenthood and partners' satisfaction with their relationship after the first child in Australia.

[34] Hitimana R, Lindholm L, Krantz G, et al. Health-related quality of life determinants among Rwandan women after delivery: does antenatal care utilization matter? A cross-sectional

For peer review only - http://bmjopen.bmj.com/site/about/guidelines.xhtml

study. DOI: 10.1186/s41043-018-0142-4.

[35] Shishehgar S, Mahmoodi A, Dolatian M, et al. The relationship of social support and quality of life with the level of stress in pregnant women using the PATH model. *Iran Red Crescent Med J* 2013; 15: 560–565.

[36] Abdollahpour S, Keramat A. The Impact of Perceived Social Support from Family and Empowerment on Maternal Wellbeing in the Postpartum Period. *J Midwifery Reprod Heal* 2016; 4: 779–787.

[37] Ruark A, Chase R, Hembling J, et al. Measuring couple relationship quality in a rural African population: Validation of a Couple Functionality Assessment Tool in Malawi. Epub ahead of print 2017. DOI: 10.1371/journal.pone.0188561.

[38] Webster J, Nicholas C, Velacott C, et al. Validation of the WHOQOL-BREF among women following childbirth. *Aust New Zeal J Obstet Gynaecol* 2010; 50: 132–137.

[39] National Institute for Health and Care Excellence N. *Intrapartum care for healthy women and babies Clinical guideline*. 2014.

[40] Mahumud RA, Ali N, Sheikh N, et al. Measuring perinatal and postpartum quality of life of women and associated factors in semi-urban Bangladesh. *Qual Life Res*. Epub ahead of print 2019. DOI: 10.1007/s11136-019-02247-0.

[41] Bahrami N, Karimian Z, Bahrami ; Somayeh, et al. Comparing the Postpartum Quality of Life Between Six to Eight Weeks and Twelve to Fourteen Weeks After Delivery in Iran. *Iran Red Crescent Med J* 2014; 16: 16985.

[42] Chinweuba AU, Okoronkwo IL, Anarado AN, et al. Differentials in health-related quality of life of employed and unemployed women with normal vaginal delivery. Epub ahead of print 2018. DOI: 10.1186/s12905-017-0481-0.

For peer review only

BMJ Open

Investigating the effect of relationship satisfaction on postpartum women’s health-related quality of life in Burkina Faso: a cross-sectional analysis

Journal:	*BMJ Open*
Manuscript ID	bmjopen-2020-048230.R1
Article Type:	Original research
Date Submitted by the Author:	24-Jul-2021
Complete List of Authors:	Lokubal, Paul; University of Oxford, Department of Population Health; London School of Hygiene & Tropical Medicine, Department of Population Health<br>Calvert, Clara; London School of Hygiene and Tropical Medicine, Department of Population Health<br>Cousens, Simon; London School of Hygiene & Tropical Medicine, Department of Infectious Disease Epidemiology<br>Daniele, Marina; King's College London, Women and Children’s Health<br>Ganaba, Rasmané; CHU-SS Bobo Dioulasso<br>Filippi, Veronique; London School of Hygiene & Tropical Medicine, Department of Infectious Disease Epidemiology
**Primary Subject Heading**:	Public health
Secondary Subject Heading:	Global health, Sexual health
Keywords:	PUBLIC HEALTH, Health policy < HEALTH SERVICES ADMINISTRATION & MANAGEMENT, SEXUAL MEDICINE

SCHOLARONE™
Manuscripts

I, the Submitting Author has the right to grant and does grant on behalf of all authors of the Work (as defined in the below author licence), an exclusive licence and/or a non-exclusive licence for contributions from authors who are: i) UK Crown employees; ii) where BMJ has agreed a CC-BY licence shall apply, and/or iii) in accordance with the terms applicable for US Federal Government officers or employees acting as part of their official duties; on a worldwide, perpetual, irrevocable, royalty-free basis to BMJ Publishing Group Ltd ("BMJ") its licensees and where the relevant Journal is co-owned by BMJ to the co-owners of the Journal, to publish the Work in this journal and any other BMJ products and to exploit all rights, as set out in our licence.

The Submitting Author accepts and understands that any supply made under these terms is made by BMJ to the Submitting Author unless you are acting as an employee on behalf of your employer or a postgraduate student of an affiliated institution which is paying any applicable article publishing charge ("APC") for Open Access articles. Where the Submitting Author wishes to make the Work available on an Open Access basis (and intends to pay the relevant APC), the terms of reuse of such Open Access shall be governed by a Creative Commons licence – details of these licences and which Creative Commons licence will apply to this Work are set out in our licence referred to above.

Other than as permitted in any relevant BMJ Author's Self Archiving Policies, I confirm this Work has not been accepted for publication elsewhere, is not being considered for publication elsewhere and does not duplicate material already published. I confirm all authors consent to publication of this Work and authorise the granting of this licence.

For peer review only

Investigating the effect of relationship satisfaction on postpartum women's health-related quality of life in Burkina Faso: a cross-sectional analysis

Corresponding author: Paul Lokubal, Doctoral Research Student, DPhil Population Health, National Perinatal Epidemiology Unit, Nuffield Department of Population Health, University of Oxford, Old Road Campus, Headington, Oxford OX3 7LF, UK
paul.lokubal@ndph.ox.ac.uk

Co-authors:

1. Clara Calvert, PhD; Department of Population Health, Faculty of Epidemiology and Population Health, The London School of Hygiene and Tropical Medicine, Keppel Street, Bloomsbury, London, WC1E 7HT, UK
2. Simon Cousens, MSc; Department of Infectious Disease Epidemiology, Faculty of Epidemiology and Population Health, The London School of Hygiene and Tropical Medicine, Keppel Street, Bloomsbury, London, WC1E 7HT, UK
3. Marina A.S. Daniele, PhD; Department of Women and Children's Health, School of Life Course Sciences, Faculty of Life Sciences and Medicine, King's College London, Becket House, London SE1 7EU, UK
4. Rasmané Ganaba, PhD; AfricSante, Bobo-Dioulasso, Burkina Faso
5. Veronique Filippi, PhD; Department of Infectious Disease Epidemiology, Faculty of Epidemiology and Population Health, The London School of Hygiene and Tropical Medicine, Keppel Street, Bloomsbury, London, WC1E 7HT, UK

Word count: 3909

Key words: quality of life; relationship satisfaction; postpartum; Burkina Faso; male partners

For peer review only

ABSTRACT

Introduction: The period following childbirth poses physiological, physical, social, and psychological challenges to women that may affect their quality of life. Few studies in Africa have explored women's health-related quality of life (HrQoL) and its determinants in postpartum populations, including the quality of women's relationships with their male partners. We investigated whether relationship satisfaction was associated with better HrQoL among postpartum women in Burkina Faso, 8 months after childbirth.

Methods: We analysed data from 547 women from the control arm of a randomized controlled trial in Burkina Faso. The study outcome was a woman's HrQoL, assessed using the cross-culturally validated World Health Organisation QOL-BREF tool, with response categories adapted for Burkina Faso. The exposure was relationship satisfaction measured using questions adapted from the Dyadic Adjustment Scale and Marital Assessment Test tools. We calculated the median HrQOL scores for the study sample, overall and for each domain of HrQOL (physical, psychological, social and environmental). The association between relationship satisfaction and HrQoL was examined using multiple linear regression models with robust standard errors.

Results: Postpartum women had high median HrQoL scores in the physical (88.1), psychological (93.1), social (86.1) and environmental (74.0) domains and overall HrQoL (84.0). We find that higher relationship satisfaction is associated with increased HrQoL. After adjusting for potential confounders, we found that for each point increase in relationship satisfaction score, the increase in HrQoL was 0.39 ($p<0.001$) for the overall HrQoL; 0.32 ($p=0.013$) for the physical domain; 0.25 ($p=0.037$) for the psychological domain; 0.46 ($p<0.001$) in the social domain; and 0.49 ($p<0.001$) for the environmental domain.

For peer review only - http://bmjopen.bmj.com/site/about/guidelines.xhtml

Conclusion: Higher relationship satisfaction is associated with higher HrQoL scores. Policies should aim to support women to cope with the challenges of childbirth and childcare in the postpartum period to improve postpartum women's HrQoL.

Strengths and Limitations

- This is one of the few studies to explore HrQoL during the extended postpartum period in an African setting, whereas most studies have documented it during pregnancy or the immediate postpartum period.
- Data collection on HrQoL and relationship satisfaction took place at one point in time eight months after childbirth and we cannot rule out reverse causality in which women who have high HrQoL scores are consequently more likely to feel satisfied with their relationship
- We did not validate the tools locally for collecting the outcomes and the main exposure although we adapted them to suit the local context and also conducted rigorous pretesting to ensure it was adequately understood by the participants

For peer review only - http://bmjopen.bmj.com/site/about/guidelines.xhtml

INTRODUCTION

The 1948 definition of health by the World Health Organisation (WHO) as "*a state of complete physical, mental and social well-being and not merely the absence of disease or infirmity*",[1] revolutionised the concept of health and led to major advances in its assessment beyond traditional health status indicators of morbidity and mortality. This definition led to the development of subjective concepts such as quality of life (QoL),[2]. According to WHO, QoL is defined as *"individuals' perceptions of their position in life in the context of the culture and value systems in which they live and in relation to their goals, expectations, standards and concerns*",[2, 3]. It is a multidimensional concept that captures all spheres of a person's life; it includes physical, mental, social and functional aspects of an individual's life and their satisfaction with it,[4]. According to Torrance, health-related quality of life (HrQoL) is "*a subset [of QoL] relating only to the health domain of that [person's] existence*",[5].

Following childbirth and during the postpartum period, many women experience physical, emotional, functional, social and psychological challenges that can affect their wellbeing,[6–10]. For example, one study of women 9-12 months postpartum in the US found that to 69% of women experienced at least one physical symptom following childbirth,[7], while another study conducted in Spain reported that 85% of women experienced fatigue six weeks after childbirth,[8]. Some of these challenges are known to persist well beyond the traditional six week postpartum period and are associated with poor overall HrQoL among postpartum women,[7, 11].

For peer review only - http://bmjopen.bmj.com/site/about/guidelines.xhtml

The reasons for poor health-related quality of life noted amongst women in the postpartum period are complex and are likely to be driven by a range of different factors including, for example, clinical experiences (i.e. if any complications occurred during delivery), and socio-demographic and economic characteristics,[11–16]. There is a growing body of evidence pointing to the important role that relationship quality plays in HrQoL for women, including during pregnancy and in the postpartum period,[16–20]. The relationship between relationship satisfaction and quality of life is complex and maybe reciprocal in which relationship satisfaction may influence or be influenced by quality of life,[21, 22]. There are many possible mechanisms through which higher relationship quality may lead to improved HrQoL among postpartum women including existence of emotional connection between spouses which helps to protect against postpartum depression,[16, 23]; easier resolution of conflicts and sharing of childcare responsibilities,[12], and social, financial and emotional support from the male partner following childbirth and during the postnatal period,[24, 25]. There are only a few, generally small, studies that have directly examined the influence of relationship satisfaction on women's postpartum HrQoL. In these studies, spousal support,[24, 25], staying with the partner postpartum,[26] and women's marital satisfaction with their spouses,[16–20] are associated with higher HrQoL among postpartum women.

Improving maternal wellbeing is a key priority with the Sustainable Development Goals agenda,[27]. Given the paucity of studies conducted in Sub-Saharan Africa on women's postpartum HrQoL and the interest in the impact of male partner involvement on women's health and wellbeing,[28], we analysed data from 547 women who were

For peer review only - http://bmjopen.bmj.com/site/about/guidelines.xhtml

enrolled in the control arm of a randomised controlled trial to investigate the effects of relationship satisfaction on postpartum women's HrQoL in Burkina Faso.

METHODS

Dataset and study design

The study is a secondary analysis of data from the control arm of an individual, non-blinded randomised-controlled trial (RCT) conducted in five primary health care facilities in three health districts in the city of Bobo-Dioulasso, Burkina Faso. The data were collected between 16th February 2015 and 4th July 2016. The details of the study design, data collection methods and data management, and ethical approval are described in detail elsewhere,[28]. In brief, the trial investigated the effectiveness of a health services intervention designed to increase male partners' involvement in maternity care, hypothesising that the intervention would increase postnatal care attendance, the duration of exclusive breastfeeding and the use of postpartum contraception[28]. Women were eligible for the trial if they were aged 15-45 years and living with a male partner – either cohabiting or legally married,[28]. The RCT intervention was provided only to named individuals who received personalised invitations to attend a series of three educational sessions in the health facility, and health workers checked the names of those attending against a list of people invited to minimise contamination between the study arms due to social interactions between individuals in the community. A total of 1114 women (583 in the intervention arm and 561 in the control group) were enrolled in the study and followed from around 20 weeks of gestation up to eight months postpartum,[28].

For peer review only - http://bmjopen.bmj.com/site/about/guidelines.xhtml

For peer review only

Ethics

The original study obtained ethics approvals from the London School of Hygiene and Tropical Medicine (LSHTM) (Reference Number 8787), Population Council (Reference Number 662), and Burkina Faso Comite D'Ethique Pour La Recherche En Sante (Reference Number Decembre 2014). This was a secondary data analysis of a clinical trial registered with ClinicalTrials.gov (NCT02309489). The ethics for this project was obtained from LSHTM MSc Ethics and Research Committee (Reference Number 16964).

Patient and public involvement

This was a secondary data analysis of a primary study and while full participant consent was obtained in the primary study, it was not required for this analysis and publication. The dataset has been published, as required by the funder (DFID) on the London School of Hygiene and Tropical Medicine Data Compass repository following full de-identification (e.g., all personal names removed, dates changed to month/year format and health centre names anonymised). Researchers can apply to use the data which can be released at the discretion of the study authors. Only the variables needed for the proposed secondary analyses are released.

Variables

Outcome: health-related quality of life

The outcome for this analysis is HrQoL and its four domains, measured at eight months postpartum [3]. Data on HrQoL were collected using the French version of the WHO

For peer review only - http://bmjopen.bmj.com/site/about/guidelines.xhtml

Quality of Life – BREF (WHOQOL-BREF) tool,[2] which has been validated among Fench-speaking populations,[29]. This tool has been validated for use in general population in Africa, but not specifically Burkina Faso, so response options were adapted to the local context by the study authors to reflect the low formal education level among women and to address difficulties in differentiating between response options in local languages,[28]. All the questions were translated to and back translated from French into Dioula and Moore during training workshops involving the principal investigator, the local field supervisor and the five interviewers. The questionnaires were then pre-tested by the interviewers with acquaintances or women in their neighbourhood. They were asked to report back on the intelligibility and cultural appropriateness of the proposed question formulation. Following their feedback, small further modifications were made.

The WHOQOL-BREF is a 26-item quality of life assessment tool covering four domains: physical (7 items – enquired about pain and discomfort; energy and fatigue; sleep and rest; dependence on medication; mobility; activities of daily living and working capacity), psychological (six items – enquired about positive feelings; negative feelings; self-esteem; thinking learning; memory and concentration; body image; spirituality, religion and personal beliefs), social (three items – personal relations; sex; and practical social support), and environmental (eight items – financial resources; information and skills; recreation and leisure; home environment; access to health and social care; physical safety and security; physical environment; and transport). There are two further questions on general quality of life and general health,[4]. The items probed women's perceptions about their life in the four weeks preceding the

For peer review only - http://bmjopen.bmj.com/site/about/guidelines.xhtml

questionnaire. For this study, each item was rated on a 3-point Likert Scale (i.e., dissatisfied/low=1, neither satisfied nor dissatisfied/moderate/average=2, satisfied/very much/high=3) with lower scores indicating lower HrQoL and the higher scores higher HrQoL. We summed these scores to give an overall HrQoL score and individual domain scores for each individual. We then transformed the respective scores to a 20-point scale and subsequently to a 100-point score scale as per WHO instructions,[3] and analysed it as a continuous variable.

Exposure: relationship satisfaction

The primary predictor for this study was relationship satisfaction measured at eight months postpartum. We defined "relationship satisfaction" in the postpartum period as a "*woman's satisfaction with her relationship with her partner and the degree of communication, shared decision-making and agreement between the couple,*[28]. Data for this variable was only collected for women who were still staying with their partners or husbands eight months after childbirth. If women became single by 8 months postpartum, data on relationship satisfaction were not collected and therefore these women are not part of the sample analysed. We calculated the relationship satisfaction score using a combination of 35 pretested questions adapted from the Marital Adjustment Test and Dyadic Adjustment Scale,[30, 31] but not locally validated for use in Burkina Faso. Women were asked about their satisfaction with their partner, satisfaction with degree of communication, satisfaction with shared decision-making and agreement on 12 questions (family finances, the woman's own family, the woman's in-laws, number of children, children's health, children's education, children's nutrition, man's work, woman's work, time the couple spent together, family planning and co-

For peer review only - http://bmjopen.bmj.com/site/about/guidelines.xhtml

spouses or other women). Lower scores on these questions indicate lower satisfaction. We summed the scores from each individual question across all the questions to generate the total score. We analysed relationship satisfaction as a continuous variable.

Potential confounders

We considered the following variables as potential confounders of the association between relationship satisfaction and HrQoL: women's age at enrolment (15-24, 25-34 and 35+); women's education level (none, primary, and secondary or higher); male partner's age at enrolment (20-29, 30-39 and 40+); male partner's education level (none, primary, secondary, and tertiary); type of marriage (polygamy and monogamy); women's parity (primiparous – first child for that childbirth, secundipara – second child for that childbirth, multipara – more than two children for that childbirth); woman's postpartum place of residence (with her parents/in-laws and with male partner); source of finance for women's health care after childbirth (divided into four categories of did not attended care, paid for herself, care was paid for by partner, and care was paid for by others); woman's need for contraception (not met and met); and woman's views on domestic violence (justified for at least one reason and not justified at all).

For peer review only - http://bmjopen.bmj.com/site/about/guidelines.xhtml

Statistical analysis

We examined the distribution of participants' characteristics at baseline and at eight months using percentages for categorical variables and means, standard deviations, medians and interquartile ranges for continuous variables. We excluded missing data as it was minimal.

We examined the internal consistency and reliability of both the WHOQOL-BREF tool and relationship satisfaction index using Cronbach's alpha coefficient, which assesses how well a set of items, variables or attributes measures a single, one-dimensional latent construct among participants involved in a study. We considered Cronbach's alpha coefficient values 0.70 and above satisfactory,[32]. We then calculated the median and interquartile ranges for the HrQoL (overall score and for each domain). This was done for the total sample of women and also stratified by relationship satisfaction. We conducted Spearman's rank correlation to investigate any correlation between the domain scores and relationship satisfaction.

In the multivariate analysis, we considered all the other variables potential confounders for the association between relationship satisfaction and HrQoL. Due to presence of heteroskedasticity, we ran a multiple linear regression models with robust standard errors to estimate adjusted regression coefficients, adding all the variables to the model simultaneously. We analysed the data using STATA/SE 15.1.

For peer review only

RESULTS

Of the 561 women enrolled in the control arm of the study, 14 were missing at the eight months' postpartum follow-up period: 11 were lost-to-follow-up without further information, one moved away, one withdrew voluntarily from the study and one died. Thus, the analysis is based on 547 women who were still enrolled in the study at eight months postpartum.

Table 1 shows women's and their partner's characteristics at baseline and at 8 months. The relationship satisfaction score ranged from 3 to 56, with a mean score of 30.5 (standard deviation (SD) = 10.1), median score of 30 and interquartile range (IQR) of 13 (24-37). The Cronbach's alpha coefficient for relationship satisfaction was 0.849 (not shown). The youngest woman was 16 years and the oldest was 43 years old. The median age of the women was 26 years with an IQR of 9 years (21-30 years) and only 64 (12%) were 35 years and older. About 50% of women had no formal education and 85% were in a monogamous relationship. There was information available on 492 male partners with a median age of 33 years (IQR 29-39 years). The youngest was 20 years and the oldest was 62 years old and about half (49%) of the men were aged 30-39 years. Over 70% (358) of the men had either no education or had attained primary education only. Eighty-five percent (457) of women stayed at the partner's place postpartum, 64% (346) had their health care paid for by their partners while 81% (438) had met need for family planning while 61% did not view domestic violence justifiable at all.

For peer review only - http://bmjopen.bmj.com/site/about/guidelines.xhtml

1 *Table 1: Demographic Characteristics of Women at the Baseline and 8 Months (number, N=547)*

Variable	Number (n)	Percent (%)	Mean	SD
**Relationship Satisfaction***	533		30.5	10.1
**Age (in years)**				
15-24	233	42.6		
25-34	250	45.7		
35+	64	11.7		
**Education level reached**				
None	271	49.5		
Primary	163	29.8		
Secondary or higher	113	20.7		
**Type of Marriage**				
Polygamous	84	15.4		
Monogamous	463	84.6		
**Woman's parity**				
Primiparous	139	25.4		
Secundipara	222	40.6		
Multiparous	186	34.0		
**Male partner's age (in years) ****				
20-29	133	27.0		
30-39	240	48.8		
40+	119	24.2		
**Male partner's education level reached*****				
None	236	47.9		
Primary	122	24.8		
Secondary	87	17.7		
Tertiary	48	9.7		
**Woman's Postpartum Place of Residence ******				
In-laws/own family	82	15.2		
Husband	457	84.8		
**Funding for woman's care******				
Did not attend care	91	16.9		
Woman	63	11.7		
Partner	346	64.2		
Others	39	7.2		
**Meet need for family planning******				
Unmet	101	18.7		
Met	438	81.3		
**Views on domestic violence******				
Justified for at least one reason	210	39.0		
Not justified at all	329	61.0		

2 ** Data on only 533 observations available ** Data on only 492 observations available*
3 **** Data on only 493 observations available **** Data on only 539 observations available*

For peer review only - http://bmjopen.bmj.com/site/about/guidelines.xhtml

Transformed HrQoL scores with Cronbach's alpha coefficient (N=547) shows transformed HrQoL scores with medians and interquartile ranges (IQR). Overall, we observed excellent internal consistency across all 26 questions with a Cronbach's alpha coefficient of 0.853. For the individual domains Cronbach's alpha was as follows:0.703 for physical, 0.721 for psychological, 0.383 for social and 0.555 for environmental. In this population, the median overall HrQoL score was 84.0 (IQR 14.4).

Table 2: Transformed HrQoL scores with Cronbach's alpha coefficient (N=547)

Domains	Number	Mean	Standard deviation	Median	25th percentile	75th percentile	Cronbach's alpha
**Physical***	546	85.0	12.9	88.1	82.1	94.0	0.703
**Psychological****	545	88.4	13.4	93.1	79.2	100	0.721
**Social****	545	88.0	14.0	86.1	86.1	100	0.383
**Environmental****	545	74.5	12.9	74.0	63.5	84.4	0.550
**Overall*****	540	83.0	10.5	84.0	76.0	90.4	0.853

1 observation missing; **2 observations missing; * 7 observations missing*

Table 3 below shows the Spearman's rank correlation results between the domains of HrQoL. We noted statistically significant moderate correlation between the physical and psychological domains (58%, p-value <0.001), physical and environmental domains (56%, p<0.001), psychological and environmental domains (58%, p-value <0.001), psychological and social domains (47%, p-value <0.001), and social and environmental domains (50%, p-value <0.001).

Table 3: Correlation matrix between HrQoL domains

	Physical	Psychological	Social	Environmental
**Physical**	1.00			
**Psychological**	0.58 (<0.001)	1.00		
**Social**	0.36 (<0.001)	0.47 (<0.001)	1.00	
**Environmental**	0.56 (<0.001)	0.58 (<0.001)	0.50 (<0.001)	1.00

**p-values in brackets.*

For peer review only - http://bmjopen.bmj.com/site/about/guidelines.xhtml

Table 4 shows the unadjusted and adjusted mean regression coefficients with robust standard errors for the association between HrQoL and relationship satisfaction. In both unadjusted and fully adjusted models, relationship adjustment was associated with the overall HrQoL and its domains. We found that for each point increase in relationship score, the overall HrQoL score increased by 0.39 (p-value <0.001) in both the unadjusted (95% CI: 0.23-0.56) and adjusted regression models (95% CI: 0.20-0.57). In the unadjusted model, the highest increases in HrQoL were noted with the social (regression coefficient =0.56, 95% CI: 0.35-0.77, p-value <0.001) and the environmental domains (regression coefficient =0.50, 95% CI: 0.30-0.69, p-value <0.001) while the lowest increases were noted with the psychological (regression coefficient =0.25, 95% CI: 0.05-0.44, p-value =0.013 and physical (regression coefficient =0.28, 95% CI: 0.07-0.49, p =0.010). There were no differences in regression coefficients between the adjusted and unadjusted models in the psychological and the environmental domains while the regression coefficient for the physical domain increased to 0.32 (95% CI: 0.07-0.57, p-value = 0.013) but that for the social domain decreased to 0.46 (95% CI: 0.22-0.69, p-value <0.001).

*Table 4: Association between Relationship Adjustment and HrQoL domains using multiple linear regression with robust standard errors**

	**Unadjusted regression coefficients**				**Adjusted regression coefficients***			
**Relationship satisfaction**	Coef.	95% CI		p-value	Coef.	95% CI		p-value
**Physical**	0.28	0.07	0.49	0.010	0.32	0.07	0.57	0.013
**Psychological**	0.25	0.05	0.44	0.013	0.25	0.02	0.49	0.037
**Social**	0.56	0.35	0.77	<0.001	0.46	0.22	0.69	<0.001
**Environment**	0.50	0.30	0.69	<0.001	0.49	0.25	0.73	<0.001

For peer review only - http://bmjopen.bmj.com/site/about/guidelines.xhtml

**Overall HrQoL**	0.39	0.23	0.56	<0.001	0.39	0.20	0.57	<0.001

**Adjusted for age, education level, type of marriage, parity, male partner's age, male partner's education level, woman's postpartum place of residence, funding for woman's health care and met need for contraception. Coef. = regression coefficient; CI = Confidence Interval.*

DISCUSSION

Key Findings

Overall, this study shows that higher relationship satisfaction scores are associated with better HrQoL among postpartum women in Burkina Faso for all the individual domains of HrQoL and the overall HrQoL

The dose-response association between relationship satisfaction and the overall HrQoL and its domains is consistent with findings from studies elsewhere that demonstrate that higher marital or relationship satisfaction is associated with an improved HrQoL among postpartum women,[11, 17, 33–36]. These findings are also consistent with findings from a systematic review exploring what matters to women in the postnatal period that showed that family relationships as well as overcoming physical and emotional challenges in the postnatal period were important considerations for women,[37]. Higher relationship satisfaction is likely to be related to women's assessments of better spousal social, economic and emotional support in the postpartum period, factors known to be associated with higher HrQoL overall,[24, 26, 38–40]. Better spousal support has been shown to lead to reductions in stress, anxiety during pregnancy, childbirth and in the postpartum period, offsetting some of the challenges of coping with parenthood and child care,[26]. In our study, we defined relationship satisfaction in relation to shared decision making, agreement on key family issues and communication between the couples. Couples who frequently discuss, agree, and make joint decisions on key family

For peer review only - http://bmjopen.bmj.com/site/about/guidelines.xhtml

issues are likely to support each other to achieve their desired targets and thus have a higher HrQoL than those who do not, [24, 25]. Increased spousal support may be responsible for the observed association between better relationship scores with HrQoL. However, women in Burkina Faso typically receive support from their extended family and social network, often in the form of financial and other assistance (such as helping in the care of the newborn) following childbirth,[9]. Thus, factors other than spousal support may explain the high HrQoL reported by women in our study. It is also plausible that a positive feedback loop or reciprocal relationship exists between relationship satisfaction and HrQoL in which relationship satisfaction influences and is influenced by HrQoL,[21, 22], in which, for example, better relationship satisfaction leads to better HrQoL which in turn leads to better relationship satisfaction.

Our results also show that overall, women had high HrQoL scores at eight months postpartum. In a study a validating the WHOQOL-BREF tool at 6 weeks postpartum among Australian postpartum women with postnatal depression (PND) and those without PND, women without PND had higher mean scores compared to those PND; mean overall score for HrQoL for women without PND was 15.8 in the 20-score scale (equivalent to 73.9 in the 100-score scale) and the highest mean score of HrQoL for women without PND was observed in the environmental domain (16.2 in the 20-score scale, equivalent to 76.3 when transformed to the 100-score scale) while the lowest was observed in the psychological scale (15.5/20 or 71.9/100),[41]. In our study, however, the highest mean score was observed in the psychological domain with the lowest obtained in the environmental domain. The differences are likely due to sociodemographic and economic differences as well as differences in infrastructure,

For peer review only - http://bmjopen.bmj.com/site/about/guidelines.xhtml

accessibility of local health-promoting resources, and amenities of the local environments between Australian women (high-income country) and those from our study (low-income country). Whereas most studies show that women's postpartum HrQoL decreases following childbirth, this period also often brings joy to women and their partners and may be associated with improved HrQoL,[42]. It is also possible that any childbirth related-health issues, for example postpartum depression, will have resolved by eight months and women may have adapted to the demands of caring for a new infant. Other studies suggest that HrQoL is related to time from childbirth. A longitudinal study comparing perinatal and postnatal HrQoL among 465 women in semi-rural Bangladesh showed that overall mean HrQoL scores increased by 37% from the perinatal period (within 2 to 7 days) to the postpartum period (within 40-60 days),[43]. The same study also showed that whereas 58% of women experienced moderate or severe levels of health problems in the perinatal period, these (health problems) resolved in the postpartum period and women experienced a high quality of life,[43]. Another prospective longitudinal study of Iranian women at 6-8 and 12-14 weeks postpartum showed that women had higher HrQoL scores in the 12-14 weeks' period compared to 6-8 weeks,[44]. In contrast, a study comparing HrQoL among employed and unemployed Nigerian women at 6, 12 and 18 weeks postpartum showed that all women had the highest HrQoL scores at 12 weeks irrespective of employment status while employed women – with only three months' maternity leave – scored lower than the unemployed women at all three timepoints, underscoring the importance of other determinants such as women's employment in addition to temporal influences on postpartum women's HrQoL,[45]. However, the duration from childbirth to the time of

For peer review only - http://bmjopen.bmj.com/site/about/guidelines.xhtml

HrQoL assessment differs markedly between women in our study (eight months) from most of the women studied.

The Cronbach's alpha coefficient was satisfactory for the overall HrQoL, and the physical and psychological domains, but low for the social and environmental domains. This may be related to the small numbers of items for the social domain, the unrelatedness of the questions to a particular domain among this population, or the heterogeneity of the questions,[2, 46, 47]. Although the internal consistency of the WHOQOL-BREF tool has been established in the general population,[2] and among postpartum women,[41] and found to be satisfactory, it has not been validated among postpartum women in Burkina Faso or Africa. Using a 3-Likert WHOQOL-BREF questionnaire on a large sample of 1,963 Nigerian adolescents, Akpa and Fowobaje showed that a two-factor model of the WHOQOL-BREF in Nigeria and related settings had better internal consistency and reliability than the current universally accepted and used four-factor model,[47]. It is thus possible that the differential conceptualisation of the WHOQOL-BREF tool questions,[47] may account for the low Cronbach's alpha in the social and environmental domains in our study population.

Study Strengths and Limitations

This is one of the few studies to explore HrQoL during the extended postpartum period in an African setting, whereas most studies have documented it during pregnancy or the immediate postpartum period.

Our study has several limitations. It was conducted in Bobo-Dioulasso, an urban setting where access to health care is relatively easy and does not therefore represent the

For peer review only - http://bmjopen.bmj.com/site/about/guidelines.xhtml

experience of women in the entire country. Data collection on HrQoL and relationship satisfaction took place at one point in time eight months after childbirth and we cannot rule out reverse causality in which women who have high HrQoL scores are consequently more likely to feel satisfied with their relationship. In addition, we did not validate the tools locally for collecting the outcomes and the main exposure although we adapted them to suit the local context and also conducted rigorous pretesting to ensure it was adequately understood by the participants. However, both tools had good internal reliability consistency (relationship satisfaction 0.849 and HrQoL 0.853) while the results for the social domain need to be interpreted with caution due to its low Cronbach's alpha. Although we did not compare the 5-rated and 3-rated Likert scales, the good internal consistency of the 3-rated Likert scale suggests that it may be preferable in situations where many variables need to be collected in a short time with resources are limited and participants have low education levels which may present semantic challenges that make it difficult to distinguish between some response options in the questionnaire. However, a validation of the WHOQOL-BREF tool for use among postpartum women in SSA is needed.

Our study may have also suffered from several response biases such as social desirability bias, satisficing, acquiescence, and positive biases. Finally, we did not include in the analysis all potential confounders of the association between relationship satisfaction and HrQoL such as sex of the babies, delivery mode and employment that are known to affect HRQOL among postpartum women. This was due to the small number of observations in some categories, the long-time lag between the occurrence of an event (such as type of delivery) and the administration of the questions, and most

For peer review only - http://bmjopen.bmj.com/site/about/guidelines.xhtml

of our study participants did not have meaningful gainful employment and as such, categorisation was difficult.

CONCLUSION

Relationship satisfaction is associated with improved HrQoL among postpartum women in Burkina Faso, an important finding for public health practitioners who seek to improve women's wellbeing after childbirth. In another study, we found that an intervention promoting male partner involvement in maternity care in the same population improved relationship satisfaction, alongside uptake of maternity care. It is therefore plausible that policies that support male involvement in maternal health might also lead to increased HrQoL directly or through relationship satisfaction. Further observational and intervention research would be needed to confirm this link and other determinants of women's HrQoL.

FUNDING

This study was funded by the Strengthening Evidence for Programming on Unintended Pregnancy (STEP UP) Research Program Consortium (grant code EPIDHC20) of which Veronique Filippi is a member, and by a contribution from the Faculty of Epidemiology and Population Health, London School of Hygiene and Tropical Medicine. STEP UP is funded by UKAid from the Department for International Development.

ACKNOWLEDGEMENTS

For peer review only

The authors wish to acknowledge the Economic and Social Research Council (ESRC) which funded Marina Daniele's studies at the London School of Hygiene and Tropical Medicine and the Chevening Scholarship and the London School of Hygiene and Tropical Medicine which funded Paul Lokubal's MSc studies at the London School of Hygiene and Tropical Medicine.

CONTRIBUTORS

MD and VF conceived the idea, planned and designed the primary study. PL wrote the first draft while SC reviewed and provided statistical input. CC, SC, MD, RG and VF edited the draft and provided critical insights. All authors have approved and contributed to the final submitted manuscript.

DATA AVAILABILITY STATEMENT

All data relevant to the analysis are uploaded as supplementary information and is with the editors. Re-use is permitted given the authors are acknowledged in any publication. Further data may be obtained by emailing MD.

COMPETING INTERESTS

The authors declare no competing interests; no support from any organisation for the submitted work; no financial relationships with any organisations that might have an interest in the submitted work in the previous 3 years; no other relationships or activities that could appear to have influenced the submitted work.

REFERENCES

For peer review only

For peer review only - http://bmjopen.bmj.com/site/about/guidelines.xhtml

[1] World Health Organization (WHO). *Constitution of the World Health Organization*. 1948.

[2] Skevington SM, Lotfy M, O'connell KA. *The World Health Organization's WHOQOL-BREF quality of life assessment: Psychometric properties and results of the international field trial. A Report from the WHOQOL Group*. 2004.

[3] World Health Organization (WHO). Introduction, Adminstration, Scoring and Generic Version of the Assessment: WHOQOL-BREF.

[4] World Health Organization (WHO). WHOQOL: Measuring Quality of Life.

[5] Torrance GW. Utility approach to measuring health-related quality of life. *J Chronic Dis* 1987; 40: 593–603.

[6] Collaborators G 2013 Daly and H. Global, regional, and national disability-adjusted life years (DALYs) for 306 diseases and injuries and healthy life expectancy (HALE) for 188 countries, 1990-2013: quantifying the epidemiological transition GBD 2013 DALYs and HALE Collaborators *. *Lancet (London, England)*. Epub ahead of print 2015. DOI: 10.1016/S0140-6736(15)61340-X.

[7] Webb DA, Bloch JR, Coyne JC, et al. Postpartum physical symptoms in new mothers: their relationship to functional limitations and emotional well-being. *Birth* 2008; 35: 179–87.

[8] Miguel Martínez-Galiano J, Hernández-Martínez A, Rodríguez-Almagro J, et al. Women's Quality of Life at 6 Weeks Postpartum: Influence of the Discomfort Present in the Puerperium. Epub ahead of print 2019. DOI: 10.3390/ijerph16020253.

[9] Storeng KT, Drabo S, Filippi V. Too poor to live? A case study of vulnerability and maternal mortality in Burkina Faso. *Glob Health Promot* 2013; 20: 33–38.

[10] Filippi V, Ganaba R, Calvert C, et al. After surgery: The effects of life-saving caesarean sections in Burkina Faso. *BMC Pregnancy Childbirth*; 15. Epub ahead of print 2015. DOI: 10.1186/s12884-015-0778-7.

[11] Saurel-cubizolles M, Romito, Lelong N, et al. Women ' s health after childbirth : a longitudinal study in. 2000; 107: 1202–1209.

[12] Gharibi M, Sanagouymoharer G, Yaghoubinia F. The Relationship between Quality of

For peer review only - http://bmjopen.bmj.com/site/about/guidelines.xhtml

Life with Marital Satisfaction in Nurses in Social Security Hospital in Zahedan. *Glob J Health Sci*; 8. Epub ahead of print 2016. DOI: 10.5539/gjhs.v8n2p178.

[13] Nussbeck F, Bodenmann G. Long-Term Prediction of Relationship Satisfaction and Stability by Stress , Coping , Communication , and Well-Being. Epub ahead of print 2014. DOI: 10.1080/10502556.2014.931767.

[14] Basharpoor S, Sheykholeslami A. The relation of marital adjustment and family functions with quality of life in women. *Eur J Psychol* 2015; 11: 432–441.

[15] Margelisch K, Schneewind KA, Violette J, et al. Marital stability, satisfaction and well-being in old age: variability and continuity in long-term continuously married older persons. *Aging Ment Heal* 2017; 21: 389–398.

[16] Mobarakabadi A, Fallahchai R, Askari M. The Relationship between Marital Satisfaction and Postpartum Depression in Women who Visited Health Centers in Bandar Abbas city. *J Appl Environ Biol Sci* 2014; 4: 120–124.

[17] Whisman MA, Davila J, Goodman SH. Relationship Adjustment, Depression, and Anxiety During Pregnancy and the Postpartum Period. Epub ahead of print 2011. DOI: 10.1037/a0023790.

[18] Tendais I, Figueiredo B, Canário C, et al. Couples' psychological adjustment to twin parenthood: mode of conception (spontaneous versus assisted reproduction) and gender differences. Epub ahead of print 2019. DOI: 10.1017/S1463423618000269.

[19] Ifeoma Odinka J, Nwoke M, Chukwuorji JC, et al. Dependent Personality, Mindful Awareness, and Marital Satisfaction and their Association with Postpartum Psychological Distress in Two Tertiary Hospitals, Southeast Nigeria. *J Health Care Poor Underserved* 2020; 31: 920–938.

[20] Gameiro S, Nazaré B, Fonseca A, et al. Changes in marital congruence and quality of life across the transition to parenthood in couples who conceived spontaneously or with assisted reproductive technologies. *Fertil Steril* 2011; 96: 1457–1462.

[21] Rostami A, Ghazinour M, Nygren L, et al. Health-related Quality of Life, Marital Satisfaction, and Social Support in Medical Staff in Iran. *Appl Res Qual Life* 2013; 8: 385–402.

For peer review only - http://bmjopen.bmj.com/site/about/guidelines.xhtml

[22] Burman B, Margolin G. Analysis of the association between marital relationships and health problems: An interactional perspective. *Psychol Bull* 1992; 112: 39–63.

[23] Odinka JI, Nwoke M, Chukwuorji JC, et al. Post-partum depression, anxiety and marital satisfaction: A perspective from Southeastern Nigeria. *South African J Psychiatry* 2018; 24: 1–8.

[24] Akbay AS, Taşçı-Duran E. How does spousal support affect women's quality of life in the postpartum period in Turkish culture? *Asian Women* 2018; 34: 29–45.

[25] Storeng KT, Akoum MS, Murray SF. This year i will not put her to work: The production/reproduction nexus in Burkina Faso. *Anthropol Med* 2013; 20: 85–97.

[26] Fontenele De Oliveira M, Parker L, Ahn H, et al. Maternal Predictors for Quality of Life during the Postpartum in Bra-zilian Mothers. *Health (Irvine Calif)* 2015; 7: 371–380.

[27] World Health Organization. Interventions for Improving Maternal and Newborn Health. *World Health*.

[28] Daniele MA, Ganaba R, Sarrassat S, et al. Involving male partners in maternity care in Burkina Faso: a randomized controlled trial. *Bull World Heal Organ* 2018; 96: 450–461.

[29] von Steinbüchel N, Lischetzke T, Gurny M, et al. Assessing quality of life in older people: Psychometric properties of the WHOQOL-BREF. *Eur J Ageing* 2006; 3: 116–122.

[30] Locke HJ, Wallace KM, Funk JL&, et al. *Marital Adjustment Test (MAT) Reference*. 2007.

[31] Spanier GB. Dyadic adjustment scale. *Journal of Sex and Marital Therapy*. Epub ahead of print 1979. DOI: 10.1080/00926237908403730.

[32] Taber KS. The Use of Cronbach's Alpha When Developing and Reporting Research Instruments in Science Education. *Res Sci Educ* 2018; 48: 1273–1296.

[33] Woolhouse H, McDonald E, Brown S. Women's experiences of sex and intimacy after childbirth: Making the adjustment to motherhood. *J Psychosom Obstet Gynecol* 2012; 33: 185–190.

[34] Marie Dyrdal G, Røysamb E, Bang Nes R, et al. Can a Happy Relationship Predict a Happy Life? A Population-Based Study of Maternal Well-Being During the Life Transition

For peer review only - http://bmjopen.bmj.com/site/about/guidelines.xhtml

of Pregnancy, Infancy, and Toddlerhood. Epub ahead of print 2010. DOI: 10.1007/s10902-010-9238-2.

[35] Midmer DK. *Family of origin, marital adjustment and the birth of the first child*. 1997.

[36] Luppi F, Carlo Alberto C. Adjustment to parenthood and partners' satisfaction with their relationship after the first child in Australia.

[37] Finlayson K, Crossland N, Bonet M, et al. What matters to women in the postnatal period: A meta-synthesis of qualitative studies. *PLoS One* 2020; 15: 1–23.

[38] Hitimana R, Lindholm L, Krantz G, et al. Health-related quality of life determinants among Rwandan women after delivery: does antenatal care utilization matter? A cross-sectional study. DOI: 10.1186/s41043-018-0142-4.

[39] Shishehgar S, Mahmoodi A, Dolatian M, et al. The relationship of social support and quality of life with the level of stress in pregnant women using the PATH model. *Iran Red Crescent Med J* 2013; 15: 560–565.

[40] Abdollahpour S, Keramat A. The Impact of Perceived Social Support from Family and Empowerment on Maternal Wellbeing in the Postpartum Period. *J Midwifery Reprod Heal* 2016; 4: 779–787.

[41] Webster J, Nicholas C, Velacott C, et al. Validation of the WHOQOL-BREF among women following childbirth. *Aust New Zeal J Obstet Gynaecol* 2010; 50: 132–137.

[42] National Institute for Health and Care Excellence N. *Intrapartum care for healthy women and babies Clinical guideline*. 2014.

[43] Mahumud RA, Ali N, Sheikh N, et al. Measuring perinatal and postpartum quality of life of women and associated factors in semi-urban Bangladesh. *Qual Life Res*. Epub ahead of print 2019. DOI: 10.1007/s11136-019-02247-0.

[44] Bahrami N, Karimian Z, Bahrami ; Somayeh, et al. Comparing the Postpartum Quality of Life Between Six to Eight Weeks and Twelve to Fourteen Weeks After Delivery in Iran. *Iran Red Crescent Med J* 2014; 16: 16985.

[45] Chinweuba AU, Okoronkwo IL, Anarado AN, et al. Differentials in health-related quality of life of employed and unemployed women with normal vaginal delivery. Epub ahead of

For peer review only - http://bmjopen.bmj.com/site/about/guidelines.xhtml

print 2018. DOI: 10.1186/s12905-017-0481-0.

[46] Tavakol M, Dennick R. Making sense of Cronbach's alpha. *Int J Med Educ* 2011; 2: 53–55.

[47] Akpa OM, Fowobaje KR. The Factor Structure of the Adapted WHO Quality of Life BREF questionnaire in a sample of adolescents in Nigeria. *Arch basic Appl Med*.

For peer review only

For peer review only - http://bmjopen.bmj.com/site/about/guidelines.xhtml

STROBE 2007 (v4) Statement—Checklist of items that should be included in reports of *cross-sectional studies*

Section/Topic	Item #	Recommendation	Reported on page #
**Title and abstract**	1	(*a*) Indicate the study's design with a commonly used term in the title or the abstract	1
		(*b*) Provide in the abstract an informative and balanced summary of what was done and what was found	2
**Introduction**			
Background/rationale	2	Explain the scientific background and rationale for the investigation being reported	4-5
Objectives	3	State specific objectives, including any prespecified hypotheses	6 (lines 1-2)
**Methods**			
Study design	4	Present key elements of study design early in the paper	6
Setting	5	Describe the setting, locations, and relevant dates, including periods of recruitment, exposure, follow-up, and data collection	6
Participants	6	(*a*) Give the eligibility criteria, and the sources and methods of selection of participants	6
Variables	7	Clearly define all outcomes, exposures, predictors, potential confounders, and effect modifiers. Give diagnostic criteria, if applicable	7-10
Data sources/ measurement	8*	For each variable of interest, give sources of data and details of methods of assessment (measurement). Describe comparability of assessment methods if there is more than one group	7-10
Bias	9	Describe any efforts to address potential sources of bias	6 (lines 15-20), 8(lines 6-12)
Study size	10	Explain how the study size was arrived at	6 (though not explained for our study)
Quantitative variables	11	Explain how quantitative variables were handled in the analyses. If applicable, describe which groupings were chosen and why	7-10
Statistical methods	12	(*a*) Describe all statistical methods, including those used to control for confounding	11
		(*b*) Describe any methods used to examine subgroups and interactions	Not done
		(*c*) Explain how missing data were addressed	11

For peer review only

		(*d*) If applicable, describe analytical methods taking account of sampling strategy	Not applicable
		(*e*) Describe any sensitivity analyses	Not done due to small missing data
**Results**			
Participants	13*	(a) Report numbers of individuals at each stage of study—eg numbers potentially eligible, examined for eligibility, confirmed eligible, included in the study, completing follow-up, and analysed	12
		(b) Give reasons for non-participation at each stage	12
		(c) Consider use of a flow diagram	Not necessary
Descriptive data	14*	(a) Give characteristics of study participants (eg demographic, clinical, social) and information on exposures and potential confounders	12
		(b) Indicate number of participants with missing data for each variable of interest	13, footnote Table 1; 14, footnote Table 2
Outcome data	15*	Report numbers of outcome events or summary measures	12
Main results	16	(*a*) Give unadjusted estimates and, if applicable, confounder-adjusted estimates and their precision (eg, 95% confidence interval). Make clear which confounders were adjusted for and why they were included	15
		(*b*) Report category boundaries when continuous variables were categorized	Not applicable
		(*c*) If relevant, consider translating estimates of relative risk into absolute risk for a meaningful time period	Not applicable
Other analyses	17	Report other analyses done—eg analyses of subgroups and interactions, and sensitivity analyses	Not done
**Discussion**			
Key results	18	Summarise key results with reference to study objectives	16
Limitations	19	Discuss limitations of the study, taking into account sources of potential bias or imprecision. Discuss both direction and magnitude of any potential bias	19-20
Interpretation	20	Give a cautious overall interpretation of results considering objectives, limitations, multiplicity of analyses, results from similar studies, and other relevant evidence	16-19
Generalisability	21	Discuss the generalisability (external validity) of the study results	19 (lines 16-18),
**Other information**			
Funding	22	Give the source of funding and the role of the funders for the present study and, if applicable, for the original study on which the present article is based	21

*Give information separately for cases and controls in case-control studies and, if applicable, for exposed and unexposed groups in cohort and cross-sectional studies.

For peer review only

Note: An Explanation and Elaboration article discusses each checklist item and gives methodological background and published examples of transparent reporting. The STROBE checklist is best used in conjunction with this article (freely available on the Web sites of PLoS Medicine at http://www.plosmedicine.org/, Annals of Internal Medicine at http://www.annals.org/, and Epidemiology at http://www.epidem.com/). Information on the STROBE Initiative is available at www.strobe-statement.org.

For peer review only

BMJ Open

Investigating the effect of relationship satisfaction on postpartum women's health-related quality of life in Burkina Faso: a cross-sectional analysis

Journal:	*BMJ Open*
Manuscript ID	bmjopen-2020-048230.R2
Article Type:	Original research
Date Submitted by the Author:	17-Aug-2021
Complete List of Authors:	Lokubal, Paul; University of Oxford, Department of Population Health; London School of Hygiene & Tropical Medicine, Department of Population Health<br>Calvert, Clara; London School of Hygiene and Tropical Medicine, Department of Population Health<br>Cousens, Simon; London School of Hygiene & Tropical Medicine, Department of Infectious Disease Epidemiology<br>Daniele, Marina; King's College London, Women and Children's Health<br>Ganaba, Rasmané; CHU-SS Bobo Dioulasso<br>Filippi, Veronique; London School of Hygiene & Tropical Medicine, Department of Infectious Disease Epidemiology
**Primary Subject Heading**:	Public health
Secondary Subject Heading:	Global health
Keywords:	PUBLIC HEALTH, Health policy < HEALTH SERVICES ADMINISTRATION & MANAGEMENT, Maternal medicine < OBSTETRICS

SCHOLARONE™
Manuscripts

I, the Submitting Author has the right to grant and does grant on behalf of all authors of the Work (as defined in the below author licence), an exclusive licence and/or a non-exclusive licence for contributions from authors who are: i) UK Crown employees; ii) where BMJ has agreed a CC-BY licence shall apply, and/or iii) in accordance with the terms applicable for US Federal Government officers or employees acting as part of their official duties; on a worldwide, perpetual, irrevocable, royalty-free basis to BMJ Publishing Group Ltd ("BMJ") its licensees and where the relevant Journal is co-owned by BMJ to the co-owners of the Journal, to publish the Work in this journal and any other BMJ products and to exploit all rights, as set out in our licence.

The Submitting Author accepts and understands that any supply made under these terms is made by BMJ to the Submitting Author unless you are acting as an employee on behalf of your employer or a postgraduate student of an affiliated institution which is paying any applicable article publishing charge ("APC") for Open Access articles. Where the Submitting Author wishes to make the Work available on an Open Access basis (and intends to pay the relevant APC), the terms of reuse of such Open Access shall be governed by a Creative Commons licence – details of these licences and which Creative Commons licence will apply to this Work are set out in our licence referred to above.

Other than as permitted in any relevant BMJ Author's Self Archiving Policies, I confirm this Work has not been accepted for publication elsewhere, is not being considered for publication elsewhere and does not duplicate material already published. I confirm all authors consent to publication of this Work and authorise the granting of this licence.

For peer review only

Investigating the effect of relationship satisfaction on postpartum women's health-related quality of life in Burkina Faso: a cross-sectional analysis

Corresponding author: Paul Lokubal, Doctoral Research Student, DPhil Population Health, National Perinatal Epidemiology Unit, Nuffield Department of Population Health, University of Oxford, Old Road Campus, Headington, Oxford OX3 7LF, UK

paul.lokubal@ndph.ox.ac.uk

Co-authors:

1. Clara Calvert, PhD; Department of Population Health, Faculty of Epidemiology and Population Health, The London School of Hygiene and Tropical Medicine, Keppel Street, Bloomsbury, London, WC1E 7HT, UK
2. Simon Cousens, MSc; Department of Infectious Disease Epidemiology, Faculty of Epidemiology and Population Health, The London School of Hygiene and Tropical Medicine, Keppel Street, Bloomsbury, London, WC1E 7HT, UK
3. Marina A.S. Daniele, PhD; Department of Women and Children's Health, School of Life Course Sciences, Faculty of Life Sciences and Medicine, King's College London, Becket House, London SE1 7EU, UK
4. Rasmané Ganaba, PhD; AfricSante, Bobo-Dioulasso, Burkina Faso
5. Veronique Filippi, PhD; Department of Infectious Disease Epidemiology, Faculty of Epidemiology and Population Health, The London School of Hygiene and Tropical Medicine, Keppel Street, Bloomsbury, London, WC1E 7HT, UK

Word count: 3909

Key words: quality of life; relationship satisfaction; postpartum; Burkina Faso; male partners

For peer review only - http://bmjopen.bmj.com/site/about/guidelines.xhtml

ABSTRACT

Introduction: The period following childbirth poses physiological, physical, social, and psychological challenges to women that may affect their quality of life. Few studies in Africa have explored women's health-related quality of life (HrQoL) and its determinants in postpartum populations, including the quality of women's relationships with their male partners. We investigated whether relationship satisfaction was associated with better HrQoL among postpartum women in Burkina Faso, 8 months after childbirth.

Methods: We analysed data from 547 women from the control arm of a randomized controlled trial in Burkina Faso. The study outcome was a woman's HrQoL, assessed using the cross-culturally validated World Health Organisation QOL-BREF tool, with response categories adapted for Burkina Faso. The exposure was relationship satisfaction measured using questions adapted from the Dyadic Adjustment Scale and Marital Assessment Test tools. We calculated the median HrQOL scores for the study sample, overall and for each domain of HrQOL (physical, psychological, social and environmental). The association between relationship satisfaction and HrQoL was examined using multiple linear regression models with robust standard errors.

Results: Postpartum women had high median HrQoL scores in the physical (88.1), psychological (93.1), social (86.1) and environmental (74.0) domains and overall HrQoL (84.0). We find that higher relationship satisfaction is associated with increased HrQoL. After adjusting for potential confounders, we found that for each point increase in relationship satisfaction score, the increase in HrQoL was 0.39 ($p<0.001$) for the overall HrQoL; 0.32 ($p=0.013$) for the physical domain; 0.25 ($p=0.037$) for the psychological domain; 0.46 ($p<0.001$) in the social domain; and 0.49 ($p<0.001$) for the environmental domain.

For peer review only - http://bmjopen.bmj.com/site/about/guidelines.xhtml

Conclusion: Higher relationship satisfaction is associated with higher HrQoL scores. Policies should aim to support women to cope with the challenges of childbirth and childcare in the postpartum period to improve postpartum women's HrQoL.

Strengths and Limitations

- This is one of the few studies to explore HrQoL during the extended postpartum period in an African setting, whereas most studies have documented it during pregnancy or the immediate postpartum period.
- Data collection on HrQoL and relationship satisfaction took place at one point in time eight months after childbirth and we cannot rule out reverse causality in which women who have high HrQoL scores are consequently more likely to feel satisfied with their relationship
- We did not validate the tools locally for collecting the outcomes and the main exposure although we adapted them to suit the local context and also conducted rigorous pretesting to ensure it was adequately understood by the participants

For peer review only - http://bmjopen.bmj.com/site/about/guidelines.xhtml

INTRODUCTION

The 1948 definition of health by the World Health Organisation (WHO) as "*a state of complete physical, mental and social well-being and not merely the absence of disease or infirmity*",[1] revolutionised the concept of health and led to major advances in its assessment beyond traditional health status indicators of morbidity and mortality. This definition led to the development of subjective concepts such as quality of life (QoL),[2]. According to WHO, QoL is defined as *"individuals' perceptions of their position in life in the context of the culture and value systems in which they live and in relation to their goals, expectations, standards and concerns*",[2, 3]. It is a multidimensional concept that captures all spheres of a person's life; it includes physical, mental, social and functional aspects of an individual's life and their satisfaction with it,[4]. According to Torrance, health-related quality of life (HrQoL) is "*a subset [of QoL] relating only to the health domain of that [person's] existence*",[5].

Following childbirth and during the postpartum period, many women experience physical, emotional, functional, social and psychological challenges that can affect their wellbeing,[6–10]. For example, one study of women 9-12 months postpartum in the US found that to 69% of women experienced at least one physical symptom following childbirth,[7], while another study conducted in Spain reported that 85% of women experienced fatigue six weeks after childbirth,[8]. Some of these challenges are known to persist well beyond the traditional six week postpartum period and are associated with poor overall HrQoL among postpartum women,[7, 11].

For peer review only - http://bmjopen.bmj.com/site/about/guidelines.xhtml

The reasons for poor health-related quality of life noted amongst women in the postpartum period are complex and are likely to be driven by a range of different factors including, for example, clinical experiences (i.e. if any complications occurred during delivery), and socio-demographic and economic characteristics,[11–16]. There is a growing body of evidence pointing to the important role that relationship quality plays in HrQoL for women, including during pregnancy and in the postpartum period,[16–20]. The relationship between relationship satisfaction and quality of life is complex and maybe reciprocal in which relationship satisfaction may influence or be influenced by quality of life,[21, 22]. There are many possible mechanisms through which higher relationship quality may lead to improved HrQoL among postpartum women including existence of emotional connection between spouses which helps to protect against postpartum depression,[16, 23]; easier resolution of conflicts and sharing of childcare responsibilities,[12], and social, financial and emotional support from the male partner following childbirth and during the postnatal period,[24, 25]. There are only a few, generally small, studies that have directly examined the influence of relationship satisfaction on women's postpartum HrQoL. In these studies, spousal support,[24, 25], staying with the partner postpartum,[26] and women's marital satisfaction with their spouses,[16–20] are associated with higher HrQoL among postpartum women.

Improving maternal wellbeing is a key priority with the Sustainable Development Goals agenda,[27]. Given the paucity of studies conducted in Sub-Saharan Africa on women's postpartum HrQoL and the interest in the impact of male partner involvement on women's health and wellbeing,[28], we analysed data from 547 women who were

For peer review only - http://bmjopen.bmj.com/site/about/guidelines.xhtml

enrolled in the control arm of a randomised controlled trial to investigate the effects of relationship satisfaction on postpartum women's HrQoL in Burkina Faso.

METHODS

Dataset and study design

The study is a secondary analysis of data from the control arm of an individual, non-blinded randomised-controlled trial (RCT) conducted in five primary health care facilities in three health districts in the city of Bobo-Dioulasso, Burkina Faso. The data were collected between 16th February 2015 and 4th July 2016. The details of the study design, data collection methods and data management, and ethical approval are described in detail elsewhere,[28]. In brief, the trial investigated the effectiveness of a health services intervention designed to increase male partners' involvement in maternity care, hypothesising that the intervention would increase postnatal care attendance, the duration of exclusive breastfeeding and the use of postpartum contraception[28]. Women were eligible for the trial if they were aged 15-45 years and living with a male partner – either cohabiting or legally married,[28]. The RCT intervention was provided only to named individuals who received personalised invitations to attend a series of three educational sessions in the health facility, and health workers checked the names of those attending against a list of people invited to minimise contamination between the study arms due to social interactions between individuals in the community. A total of 1114 women (583 in the intervention arm and 561 in the control group) were enrolled in the study and followed from around 20 weeks of gestation up to eight months postpartum,[28].

For peer review only - http://bmjopen.bmj.com/site/about/guidelines.xhtml

For peer review only

Ethics

The original study obtained ethics approvals from the London School of Hygiene and Tropical Medicine (LSHTM) (Reference Number 8787), Population Council (Reference Number 662), and Burkina Faso Comite D'Ethique Pour La Recherche En Sante (Reference Number Decembre 2014). This was a secondary data analysis of a clinical trial registered with ClinicalTrials.gov (NCT02309489). The ethics for this project was obtained from LSHTM MSc Ethics and Research Committee (Reference Number 16964).

Patient and public involvement

This was a secondary data analysis of a primary study and while full participant consent was obtained in the primary study, it was not required for this analysis and publication. The dataset has been published, as required by the funder (DFID) on the London School of Hygiene and Tropical Medicine Data Compass repository following full de-identification (e.g., all personal names removed, dates changed to month/year format and health centre names anonymised). Researchers can apply to use the data which can be released at the discretion of the study authors. Only the variables needed for the proposed secondary analyses are released.

Variables

Outcome: health-related quality of life

The outcome for this analysis is HrQoL and its four domains, measured at eight months postpartum [3]. Data on HrQoL were collected using the French version of the WHO

For peer review only - http://bmjopen.bmj.com/site/about/guidelines.xhtml

Quality of Life – BREF (WHOQOL-BREF) tool,[2] which has been validated among Fench-speaking populations,[29]. This tool has been validated for use in general population in Africa, but not specifically Burkina Faso, so response options were adapted to the local context by the study authors to reflect the low formal education level among women and to address difficulties in differentiating between response options in local languages,[28]. All the questions were translated to and back translated from French into Dioula and Moore during training workshops involving the principal investigator, the local field supervisor and the five interviewers. The questionnaires were then pre-tested by the interviewers with acquaintances or women in their neighbourhood. They were asked to report back on the intelligibility and cultural appropriateness of the proposed question formulation. Following their feedback, small further modifications were made.

The WHOQOL-BREF is a 26-item quality of life assessment tool covering four domains: physical (7 items – enquired about pain and discomfort; energy and fatigue; sleep and rest; dependence on medication; mobility; activities of daily living and working capacity), psychological (six items – enquired about positive feelings; negative feelings; self-esteem; thinking learning; memory and concentration; body image; spirituality, religion and personal beliefs), social (three items – personal relations; sex; and practical social support), and environmental (eight items – financial resources; information and skills; recreation and leisure; home environment; access to health and social care; physical safety and security; physical environment; and transport). There are two further questions on general quality of life and general health,[4]. The items probed women's perceptions about their life in the four weeks preceding the

For peer review only - http://bmjopen.bmj.com/site/about/guidelines.xhtml

questionnaire. For this study, each item was rated on a 3-point Likert Scale (i.e., dissatisfied/low=1, neither satisfied nor dissatisfied/moderate/average=2, satisfied/very much/high=3) with lower scores indicating lower HrQoL and the higher scores higher HrQoL. We summed these scores to give an overall HrQoL score and individual domain scores for each individual. We then transformed the respective scores to a 20-point scale and subsequently to a 100-point score scale as per WHO instructions,[3] and analysed it as a continuous variable.

Exposure: relationship satisfaction

The primary predictor for this study was relationship satisfaction measured at eight months postpartum. We defined "relationship satisfaction" in the postpartum period as a "*woman's satisfaction with her relationship with her partner and the degree of communication, shared decision-making and agreement between the couple,*[28]. Data for this variable was only collected for women who were still staying with their partners or husbands eight months after childbirth. If women became single by 8 months postpartum, data on relationship satisfaction were not collected and therefore these women are not part of the sample analysed. We calculated the relationship satisfaction score using a combination of 35 pretested questions adapted from the Marital Adjustment Test and Dyadic Adjustment Scale,[30, 31] but not locally validated for use in Burkina Faso. Women were asked about their satisfaction with their partner, satisfaction with degree of communication, satisfaction with shared decision-making and agreement on 12 questions (family finances, the woman's own family, the woman's in-laws, number of children, children's health, children's education, children's nutrition, man's work, woman's work, time the couple spent together, family planning and co-

For peer review only - http://bmjopen.bmj.com/site/about/guidelines.xhtml

spouses or other women). Lower scores on these questions indicate lower satisfaction. We summed the scores from each individual question across all the questions to generate the total score. We analysed relationship satisfaction as a continuous variable.

Potential confounders

We considered the following variables as potential confounders of the association between relationship satisfaction and HrQoL: women's age at enrolment (15-24, 25-34 and 35+); women's education level (none, primary, and secondary or higher); male partner's age at enrolment (20-29, 30-39 and 40+); male partner's education level (none, primary, secondary, and tertiary); type of relationship (polygamy and monogamy); women's parity (primiparous – first child for that childbirth, secundipara – second child for that childbirth, multipara – more than two children for that childbirth); woman's postpartum place of residence (with her parents/in-laws and with male partner); source of finance for women's health care after childbirth (divided into four categories of did not attended care, paid for herself, care was paid for by partner, and care was paid for by others); woman's need for contraception (not met and met); and woman's views on domestic violence (justified for at least one reason and not justified at all).

For peer review only

For peer review only - http://bmjopen.bmj.com/site/about/guidelines.xhtml

Statistical analysis

We examined the distribution of participants' characteristics at baseline and at eight months using percentages for categorical variables and means, standard deviations, medians and interquartile ranges for continuous variables. We excluded missing data as it was minimal.

We examined the internal consistency and reliability of both the WHOQOL-BREF tool and relationship satisfaction index using Cronbach's alpha coefficient, which assesses how well a set of items, variables or attributes measures a single, one-dimensional latent construct among participants involved in a study. We considered Cronbach's alpha coefficient values 0.70 and above satisfactory,[32]. We then calculated the median and interquartile ranges for the HrQoL (overall score and for each domain). This was done for the total sample of women and also stratified by relationship satisfaction. We conducted Spearman's rank correlation to investigate any correlation between the domain scores and relationship satisfaction.

In the multivariate analysis, we considered all the other variables potential confounders for the association between relationship satisfaction and HrQoL. Due to presence of heteroskedasticity, we ran a multiple linear regression model with robust standard errors to estimate adjusted regression coefficients, adding all the variables to the model simultaneously. We analysed the data using STATA/SE 15.1.

For peer review only - http://bmjopen.bmj.com/site/about/guidelines.xhtml

RESULTS

Of the 561 women enrolled in the control arm of the study, 14 were missing at the eight months' postpartum follow-up period: 11 were lost-to-follow-up without further information, one moved away, one withdrew voluntarily from the study and one died. Thus, the analysis is based on 547 women who were still enrolled in the study at eight months postpartum.

Table 1 shows women's and their partner's characteristics at baseline and at 8 months. The relationship satisfaction score ranged from 3 to 56, with a mean score of 30.5 (standard deviation (SD) = 10.1), median score of 30 and interquartile range (IQR) of 13 (24-37). The Cronbach's alpha coefficient for relationship satisfaction was 0.849 (not shown). The youngest woman was 16 years and the oldest was 43 years old. The median age of the women was 26 years with an IQR of 9 years (21-30 years) and only 64 (12%) were 35 years and older. About 50% of women had no formal education and 85% were in a monogamous relationship. There was information available on 492 male partners with a median age of 33 years (IQR 29-39 years). The youngest was 20 years and the oldest was 62 years old and about half (49%) of the men were aged 30-39 years. Over 70% (358) of the men had either no education or had attained primary education only. Eighty-five percent (457) of women stayed at the partner's place postpartum, 64% (346) had their health care paid for by their partners while 81% (438) had met need for family planning while 61% did not view domestic violence justifiable at all.

For peer review only - http://bmjopen.bmj.com/site/about/guidelines.xhtml

1 *Table 1: Demographic characteristics of women at the baseline and at eight months (number, N=547)*

Variable	Number (n)	Percent (%)	Mean	SD
**Relationship Satisfaction***	533		30.5	10.1
**Age (in years)**				
15-24	233	42.6		
25-34	250	45.7		
35+	64	11.7		
**Education level reached**				
None	271	49.5		
Primary	163	29.8		
Secondary or higher	113	20.7		
**Type of relationship**				
Polygamous	84	15.4		
Monogamous	463	84.6		
**Woman's parity**				
Primiparous	139	25.4		
Secundipara	222	40.6		
Multiparous	186	34.0		
**Male partner's age (in years) ****				
20-29	133	27.0		
30-39	240	48.8		
40+	119	24.2		
**Male partner's education level reached*****				
None	236	47.9		
Primary	122	24.8		
Secondary	87	17.7		
Tertiary	48	9.7		
**Woman's Postpartum Place of Residence *******				
In-laws/own family	82	15.2		
Husband	457	84.8		
**Funding for woman's care******				
Did not attend care	91	16.9		
Woman	63	11.7		
Partner	346	64.2		
Others	39	7.2		
**Meet need for family planning******				
Unmet	101	18.7		
Met	438	81.3		
**Views on domestic violence******				
Justified for at least one reason	210	39.0		
Not justified at all	329	61.0		

2 ** Data on only 533 observations available ** Data on only 492 observations available*
3 **** Data on only 493 observations available **** Data on only 539 observations available*

For peer review only - http://bmjopen.bmj.com/site/about/guidelines.xhtml

Transformed HrQoL scores with Cronbach's alpha coefficient (N=547) shows transformed HrQoL scores with medians and interquartile ranges (IQR). Overall, we observed excellent internal consistency across all 26 questions with a Cronbach's alpha coefficient of 0.853. For the individual domains Cronbach's alpha was as follows:0.703 for physical, 0.721 for psychological, 0.383 for social and 0.555 for environmental. In this population, the median overall HrQoL score was 84.0 (IQR 14.4).

Table 2: Transformed HrQoL scores with Cronbach's alpha coefficient (N=547)

Domains	Number	Mean	Standard deviation	Median	25th percentile	75th percentile	Cronbach's alpha
**Physical***	546	85.0	12.9	88.1	82.1	94.0	0.703
**Psychological****	545	88.4	13.4	93.1	79.2	100	0.721
**Social****	545	88.0	14.0	86.1	86.1	100	0.383
**Environmental****	545	74.5	12.9	74.0	63.5	84.4	0.550
**Overall*****	540	83.0	10.5	84.0	76.0	90.4	0.853

1 observation missing; **2 observations missing; * 7 observations missing*

Table 3 below shows the Spearman's rank correlation results between the domains of HrQoL. We noted statistically significant moderate correlation between the physical and psychological domains (58%, p-value <0.001), physical and environmental domains (56%, p<0.001), psychological and environmental domains (58%, p-value <0.001), psychological and social domains (47%, p-value <0.001), and social and environmental domains (50%, p-value <0.001).

Table 3: Correlation matrix between HrQoL domains

	Physical	Psychological	Social	Environmental
**Physical**	1.00			
**Psychological**	0.58 (<0.001)	1.00		
**Social**	0.36 (<0.001)	0.47 (<0.001)	1.00	
**Environmental**	0.56 (<0.001)	0.58 (<0.001)	0.50 (<0.001)	1.00

**p-values in brackets.*

For peer review only - http://bmjopen.bmj.com/site/about/guidelines.xhtml

Table 4 shows the unadjusted and adjusted mean regression coefficients with robust standard errors for the association between HrQoL and relationship satisfaction. In both unadjusted and fully adjusted models, relationship adjustment was associated with the overall HrQoL and its domains. We found that for each point increase in relationship score, the overall HrQoL score increased by 0.39 (p-value <0.001) in both the unadjusted (95% CI: 0.23-0.56) and adjusted regression models (95% CI: 0.20-0.57). In the unadjusted model, the highest increases in HrQoL were noted with the social (regression coefficient =0.56, 95% CI: 0.35-0.77, p-value <0.001) and the environmental domains (regression coefficient =0.50, 95% CI: 0.30-0.69, p-value <0.001) while the lowest increases were noted with the psychological (regression coefficient =0.25, 95% CI: 0.05-0.44, p-value =0.013 and physical (regression coefficient =0.28, 95% CI: 0.07-0.49, p =0.010). There were no differences in regression coefficients between the adjusted and unadjusted models in the psychological and the environmental domains while the regression coefficient for the physical domain increased to 0.32 (95% CI: 0.07-0.57, p-value = 0.013) but that for the social domain decreased to 0.46 (95% CI: 0.22-0.69, p-value <0.001).

*Table 4: Association between relationship satisfaction and HrQoL using multiple linear regression with robust standard errors**

	**Unadjusted regression coefficients**				**Adjusted regression coefficients***			
**Relationship satisfaction**	Coef.	95% CI		p-value	Coef.	95% CI		p-value
**Physical**	0.28	0.07	0.49	0.010	0.32	0.07	0.57	0.013
**Psychological**	0.25	0.05	0.44	0.013	0.25	0.02	0.49	0.037
**Social**	0.56	0.35	0.77	<0.001	0.46	0.22	0.69	<0.001
**Environment**	0.50	0.30	0.69	<0.001	0.49	0.25	0.73	<0.001

For peer review only - http://bmjopen.bmj.com/site/about/guidelines.xhtml

**Overall HrQoL**	0.39	0.23	0.56	<0.001	0.39	0.20	0.57	<0.001

**Adjusted for age, education level, type of relationship, parity, male partner's age, male partner's education level, woman's postpartum place of residence, funding for woman's health care, met need for contraception and views on domestic violence. Coef. = regression coefficient; CI = Confidence Interval.*

DISCUSSION

Key Findings

Overall, this study shows that higher relationship satisfaction scores are associated with better HrQoL among postpartum women in Burkina Faso for all the individual domains of HrQoL and the overall HrQoL

The dose-response association between relationship satisfaction and the overall HrQoL and its domains is consistent with findings from studies elsewhere that demonstrate that higher marital or relationship satisfaction is associated with an improved HrQoL among postpartum women,[11, 17, 33–36]. These findings are also consistent with findings from a systematic review exploring what matters to women in the postnatal period that showed that family relationships as well as overcoming physical and emotional challenges in the postnatal period were important considerations for women,[37]. Higher relationship satisfaction is likely to be related to women's assessments of better spousal social, economic and emotional support in the postpartum period, factors known to be associated with higher HrQoL overall,[24, 26, 38–40]. Better spousal support has been shown to lead to reductions in stress, anxiety during pregnancy, childbirth and in the postpartum period, offsetting some of the challenges of coping with parenthood and child care,[26]. In our study, we defined relationship satisfaction in relation to shared decision making, agreement on key family issues and communication between the couples. Couples who frequently discuss, agree, and make joint decisions on key family

For peer review only - http://bmjopen.bmj.com/site/about/guidelines.xhtml

issues are likely to support each other to achieve their desired targets and thus have a higher HrQoL than those who do not, [24, 25]. Increased spousal support may be responsible for the observed association between better relationship scores with HrQoL. However, women in Burkina Faso typically receive support from their extended family and social network, often in the form of financial and other assistance (such as helping in the care of the newborn) following childbirth,[9]. Thus, factors other than spousal support may explain the high HrQoL reported by women in our study. It is also plausible that a positive feedback loop or reciprocal relationship exists between relationship satisfaction and HrQoL in which relationship satisfaction influences and is influenced by HrQoL,[21, 22], in which, for example, better relationship satisfaction leads to better HrQoL which in turn leads to better relationship satisfaction.

Our results also show that overall, women had high HrQoL scores at eight months postpartum. In a study a validating the WHOQOL-BREF tool at 6 weeks postpartum among Australian postpartum women with postnatal depression (PND) and those without PND, women without PND had higher mean scores compared to those PND; mean overall score for HrQoL for women without PND was 15.8 in the 20-score scale (equivalent to 73.9 in the 100-score scale) and the highest mean score of HrQoL for women without PND was observed in the environmental domain (16.2 in the 20-score scale, equivalent to 76.3 when transformed to the 100-score scale) while the lowest was observed in the psychological scale (15.5/20 or 71.9/100),[41]. In our study, however, the highest mean score was observed in the psychological domain with the lowest obtained in the environmental domain. The differences are likely due to sociodemographic and economic differences as well as differences in infrastructure,

For peer review only - http://bmjopen.bmj.com/site/about/guidelines.xhtml

accessibility of local health-promoting resources, and amenities of the local environments between Australian women (high-income country) and those from our study (low-income country). Whereas most studies show that women's postpartum HrQoL decreases following childbirth, this period also often brings joy to women and their partners and may be associated with improved HrQoL,[42]. It is also possible that any childbirth related-health issues, for example postpartum depression, will have resolved by eight months and women may have adapted to the demands of caring for a new infant. Other studies suggest that HrQoL is related to time from childbirth. A longitudinal study comparing perinatal and postnatal HrQoL among 465 women in semi-rural Bangladesh showed that overall mean HrQoL scores increased by 37% from the perinatal period (within 2 to 7 days) to the postpartum period (within 40-60 days),[43]. The same study also showed that whereas 58% of women experienced moderate or severe levels of health problems in the perinatal period, these (health problems) resolved in the postpartum period and women experienced a high quality of life,[43]. Another prospective longitudinal study of Iranian women at 6-8 and 12-14 weeks postpartum showed that women had higher HrQoL scores in the 12-14 weeks' period compared to 6-8 weeks,[44]. In contrast, a study comparing HrQoL among employed and unemployed Nigerian women at 6, 12 and 18 weeks postpartum showed that all women had the highest HrQoL scores at 12 weeks irrespective of employment status while employed women – with only three months' maternity leave – scored lower than the unemployed women at all three timepoints, underscoring the importance of other determinants such as women's employment in addition to temporal influences on postpartum women's HrQoL,[45]. However, the duration from childbirth to the time of

For peer review only - http://bmjopen.bmj.com/site/about/guidelines.xhtml

HrQoL assessment differs markedly between women in our study (eight months) from most of the women studied.

The Cronbach's alpha coefficient was satisfactory for the overall HrQoL, and the physical and psychological domains, but low for the social and environmental domains. This may be related to the small numbers of items for the social domain, the unrelatedness of the questions to a particular domain among this population, or the heterogeneity of the questions,[2, 46, 47]. Although the internal consistency of the WHOQOL-BREF tool has been established in the general population,[2] and among postpartum women,[41] and found to be satisfactory, it has not been validated among postpartum women in Burkina Faso or Africa. Using a 3-Likert WHOQOL-BREF questionnaire on a large sample of 1,963 Nigerian adolescents, Akpa and Fowobaje showed that a two-factor model of the WHOQOL-BREF in Nigeria and related settings had better internal consistency and reliability than the current universally accepted and used four-factor model,[47]. It is thus possible that the differential conceptualisation of the WHOQOL-BREF tool questions,[47] may account for the low Cronbach's alpha in the social and environmental domains in our study population.

Study Strengths and Limitations

This is one of the few studies to explore HrQoL during the extended postpartum period in an African setting, whereas most studies have documented it during pregnancy or the immediate postpartum period.

Our study has several limitations. It was conducted in Bobo-Dioulasso, an urban setting where access to health care is relatively easy and does not therefore represent the

For peer review only - http://bmjopen.bmj.com/site/about/guidelines.xhtml

experience of women in the entire country. Data collection on HrQoL and relationship satisfaction took place at one point in time eight months after childbirth and we cannot rule out reverse causality in which women who have high HrQoL scores are consequently more likely to feel satisfied with their relationship. In addition, we did not validate the tools locally for collecting the outcomes and the main exposure although we adapted them to suit the local context and also conducted rigorous pretesting to ensure it was adequately understood by the participants. However, both tools had good internal reliability consistency (relationship satisfaction 0.849 and HrQoL 0.853) while the results for the social domain need to be interpreted with caution due to its low Cronbach's alpha. Although we did not compare the 5-rated and 3-rated Likert scales, the good internal consistency of the 3-rated Likert scale suggests that it may be preferable in situations where many variables need to be collected in a short time with resources are limited and participants have low education levels which may present semantic challenges that make it difficult to distinguish between some response options in the questionnaire. However, a validation of the WHOQOL-BREF tool for use among postpartum women in SSA is needed.

Our study may have also suffered from several response biases such as social desirability bias, satisficing, acquiescence, and positive biases. Finally, we did not include in the analysis all potential confounders of the association between relationship satisfaction and HrQoL such as sex of the babies, delivery mode and employment that are known to affect HRQOL among postpartum women. This was due to the small number of observations in some categories, the long-time lag between the occurrence of an event (such as type of delivery) and the administration of the questions, and most

For peer review only - http://bmjopen.bmj.com/site/about/guidelines.xhtml

of our study participants did not have meaningful gainful employment and as such, categorisation was difficult.

CONCLUSION

Relationship satisfaction is associated with improved HrQoL among postpartum women in Burkina Faso, an important finding for public health practitioners who seek to improve women's wellbeing after childbirth. In another study, we found that an intervention promoting male partner involvement in maternity care in the same population improved relationship satisfaction, alongside uptake of maternity care. It is therefore plausible that policies that support male involvement in maternal health might also lead to increased HrQoL directly or through relationship satisfaction. Further observational and intervention research would be needed to confirm this link and other determinants of women's HrQoL.

FUNDING

This study was funded by the Strengthening Evidence for Programming on Unintended Pregnancy (STEP UP) Research Program Consortium (grant code EPIDHC20) of which Veronique Filippi is a member, and by a contribution from the Faculty of Epidemiology and Population Health, London School of Hygiene and Tropical Medicine. STEP UP is funded by UKAid from the Department for International Development.

ACKNOWLEDGEMENTS

For peer review only - http://bmjopen.bmj.com/site/about/guidelines.xhtml

The authors wish to acknowledge the Economic and Social Research Council (ESRC) which funded Marina Daniele's studies at the London School of Hygiene and Tropical Medicine and the Chevening Scholarship and the London School of Hygiene and Tropical Medicine which funded Paul Lokubal's MSc studies at the London School of Hygiene and Tropical Medicine.

CONTRIBUTORS

MD and VF conceived the idea, planned and designed the primary study. PL wrote the first draft while SC reviewed and provided statistical input. CC, SC, MD, RG and VF edited the draft and provided critical insights. All authors have approved and contributed to the final submitted manuscript.

DATA AVAILABILITY STATEMENT

All data relevant to the analysis are uploaded as supplementary information and is with the editors. Re-use is permitted given the authors are acknowledged in any publication. Further data may be obtained by emailing MD.

COMPETING INTERESTS

The authors declare no competing interests; no support from any organisation for the submitted work; no financial relationships with any organisations that might have an interest in the submitted work in the previous 3 years; no other relationships or activities that could appear to have influenced the submitted work.

REFERENCES

For peer review only

For peer review only - http://bmjopen.bmj.com/site/about/guidelines.xhtml

[1] World Health Organization (WHO). *Constitution of the World Health Organization*. 1948.

[2] Skevington SM, Lotfy M, O'connell KA. *The World Health Organization's WHOQOL-BREF quality of life assessment: Psychometric properties and results of the international field trial. A Report from the WHOQOL Group*. 2004.

[3] World Health Organization (WHO). Introduction, Adminstration, Scoring and Generic Version of the Assessment: WHOQOL-BREF.

[4] World Health Organization (WHO). WHOQOL: Measuring Quality of Life.

[5] Torrance GW. Utility approach to measuring health-related quality of life. *J Chronic Dis* 1987; 40: 593–603.

[6] Collaborators G 2013 Daly and H. Global, regional, and national disability-adjusted life years (DALYs) for 306 diseases and injuries and healthy life expectancy (HALE) for 188 countries, 1990-2013: quantifying the epidemiological transition GBD 2013 DALYs and HALE Collaborators *. *Lancet (London, England)*. Epub ahead of print 2015. DOI: 10.1016/S0140-6736(15)61340-X.

[7] Webb DA, Bloch JR, Coyne JC, et al. Postpartum physical symptoms in new mothers: their relationship to functional limitations and emotional well-being. *Birth* 2008; 35: 179–87.

[8] Miguel Martínez-Galiano J, Hernández-Martínez A, Rodríguez-Almagro J, et al. Women's Quality of Life at 6 Weeks Postpartum: Influence of the Discomfort Present in the Puerperium. Epub ahead of print 2019. DOI: 10.3390/ijerph16020253.

[9] Storeng KT, Drabo S, Filippi V. Too poor to live? A case study of vulnerability and maternal mortality in Burkina Faso. *Glob Health Promot* 2013; 20: 33–38.

[10] Filippi V, Ganaba R, Calvert C, et al. After surgery: The effects of life-saving caesarean sections in Burkina Faso. *BMC Pregnancy Childbirth*; 15. Epub ahead of print 2015. DOI: 10.1186/s12884-015-0778-7.

[11] Saurel-cubizolles M, Romito, Lelong N, et al. Women ' s health after childbirth : a longitudinal study in. 2000; 107: 1202–1209.

[12] Gharibi M, Sanagouymoharer G, Yaghoubinia F. The Relationship between Quality of

For peer review only - http://bmjopen.bmj.com/site/about/guidelines.xhtml

Life with Marital Satisfaction in Nurses in Social Security Hospital in Zahedan. *Glob J Health Sci*; 8. Epub ahead of print 2016. DOI: 10.5539/gjhs.v8n2p178.

[13] Nussbeck F, Bodenmann G. Long-Term Prediction of Relationship Satisfaction and Stability by Stress , Coping , Communication , and Well-Being. Epub ahead of print 2014. DOI: 10.1080/10502556.2014.931767.

[14] Basharpoor S, Sheykholeslami A. The relation of marital adjustment and family functions with quality of life in women. *Eur J Psychol* 2015; 11: 432–441.

[15] Margelisch K, Schneewind KA, Violette J, et al. Marital stability, satisfaction and well-being in old age: variability and continuity in long-term continuously married older persons. *Aging Ment Heal* 2017; 21: 389–398.

[16] Mobarakabadi A, Fallahchai R, Askari M. The Relationship between Marital Satisfaction and Postpartum Depression in Women who Visited Health Centers in Bandar Abbas city. *J Appl Environ Biol Sci* 2014; 4: 120–124.

[17] Whisman MA, Davila J, Goodman SH. Relationship Adjustment, Depression, and Anxiety During Pregnancy and the Postpartum Period. Epub ahead of print 2011. DOI: 10.1037/a0023790.

[18] Tendais I, Figueiredo B, Canário C, et al. Couples' psychological adjustment to twin parenthood: mode of conception (spontaneous versus assisted reproduction) and gender differences. Epub ahead of print 2019. DOI: 10.1017/S1463423618000269.

[19] Ifeoma Odinka J, Nwoke M, Chukwuorji JC, et al. Dependent Personality, Mindful Awareness, and Marital Satisfaction and their Association with Postpartum Psychological Distress in Two Tertiary Hospitals, Southeast Nigeria. *J Health Care Poor Underserved* 2020; 31: 920–938.

[20] Gameiro S, Nazaré B, Fonseca A, et al. Changes in marital congruence and quality of life across the transition to parenthood in couples who conceived spontaneously or with assisted reproductive technologies. *Fertil Steril* 2011; 96: 1457–1462.

[21] Rostami A, Ghazinour M, Nygren L, et al. Health-related Quality of Life, Marital Satisfaction, and Social Support in Medical Staff in Iran. *Appl Res Qual Life* 2013; 8: 385–402.

For peer review only - http://bmjopen.bmj.com/site/about/guidelines.xhtml

[22] Burman B, Margolin G. Analysis of the association between marital relationships and health problems: An interactional perspective. *Psychol Bull* 1992; 112: 39–63.

[23] Odinka JI, Nwoke M, Chukwuorji JC, et al. Post-partum depression, anxiety and marital satisfaction: A perspective from Southeastern Nigeria. *South African J Psychiatry* 2018; 24: 1–8.

[24] Akbay AS, Taşçı-Duran E. How does spousal support affect women's quality of life in the postpartum period in Turkish culture? *Asian Women* 2018; 34: 29–45.

[25] Storeng KT, Akoum MS, Murray SF. This year i will not put her to work: The production/reproduction nexus in Burkina Faso. *Anthropol Med* 2013; 20: 85–97.

[26] Fontenele De Oliveira M, Parker L, Ahn H, et al. Maternal Predictors for Quality of Life during the Postpartum in Bra-zilian Mothers. *Health (Irvine Calif)* 2015; 7: 371–380.

[27] World Health Organization. Interventions for Improving Maternal and Newborn Health. *World Health*.

[28] Daniele MA, Ganaba R, Sarrassat S, et al. Involving male partners in maternity care in Burkina Faso: a randomized controlled trial. *Bull World Heal Organ* 2018; 96: 450–461.

[29] von Steinbüchel N, Lischetzke T, Gurny M, et al. Assessing quality of life in older people: Psychometric properties of the WHOQOL-BREF. *Eur J Ageing* 2006; 3: 116–122.

[30] Locke HJ, Wallace KM, Funk JL&, et al. *Marital Adjustment Test (MAT) Reference*. 2007.

[31] Spanier GB. Dyadic adjustment scale. *Journal of Sex and Marital Therapy*. Epub ahead of print 1979. DOI: 10.1080/00926237908403730.

[32] Taber KS. The Use of Cronbach's Alpha When Developing and Reporting Research Instruments in Science Education. *Res Sci Educ* 2018; 48: 1273–1296.

[33] Woolhouse H, McDonald E, Brown S. Women's experiences of sex and intimacy after childbirth: Making the adjustment to motherhood. *J Psychosom Obstet Gynecol* 2012; 33: 185–190.

[34] Marie Dyrdal G, Røysamb E, Bang Nes R, et al. Can a Happy Relationship Predict a Happy Life? A Population-Based Study of Maternal Well-Being During the Life Transition

For peer review only

of Pregnancy, Infancy, and Toddlerhood. Epub ahead of print 2010. DOI: 10.1007/s10902-010-9238-2.

[35] Midmer DK. *Family of origin, marital adjustment and the birth of the first child*. 1997.

[36] Luppi F, Carlo Alberto C. Adjustment to parenthood and partners' satisfaction with their relationship after the first child in Australia.

[37] Finlayson K, Crossland N, Bonet M, et al. What matters to women in the postnatal period: A meta-synthesis of qualitative studies. *PLoS One* 2020; 15: 1–23.

[38] Hitimana R, Lindholm L, Krantz G, et al. Health-related quality of life determinants among Rwandan women after delivery: does antenatal care utilization matter? A cross-sectional study. DOI: 10.1186/s41043-018-0142-4.

[39] Shishehgar S, Mahmoodi A, Dolatian M, et al. The relationship of social support and quality of life with the level of stress in pregnant women using the PATH model. *Iran Red Crescent Med J* 2013; 15: 560–565.

[40] Abdollahpour S, Keramat A. The Impact of Perceived Social Support from Family and Empowerment on Maternal Wellbeing in the Postpartum Period. *J Midwifery Reprod Heal* 2016; 4: 779–787.

[41] Webster J, Nicholas C, Velacott C, et al. Validation of the WHOQOL-BREF among women following childbirth. *Aust New Zeal J Obstet Gynaecol* 2010; 50: 132–137.

[42] National Institute for Health and Care Excellence N. *Intrapartum care for healthy women and babies Clinical guideline*. 2014.

[43] Mahumud RA, Ali N, Sheikh N, et al. Measuring perinatal and postpartum quality of life of women and associated factors in semi-urban Bangladesh. *Qual Life Res*. Epub ahead of print 2019. DOI: 10.1007/s11136-019-02247-0.

[44] Bahrami N, Karimian Z, Bahrami ; Somayeh, et al. Comparing the Postpartum Quality of Life Between Six to Eight Weeks and Twelve to Fourteen Weeks After Delivery in Iran. *Iran Red Crescent Med J* 2014; 16: 16985.

[45] Chinweuba AU, Okoronkwo IL, Anarado AN, et al. Differentials in health-related quality of life of employed and unemployed women with normal vaginal delivery. Epub ahead of

For peer review only

print 2018. DOI: 10.1186/s12905-017-0481-0.

[46] Tavakol M, Dennick R. Making sense of Cronbach's alpha. *Int J Med Educ* 2011; 2: 53–55.

[47] Akpa OM, Fowobaje KR. The Factor Structure of the Adapted WHO Quality of Life BREF questionnaire in a sample of adolescents in Nigeria. *Arch basic Appl Med*.

For peer review only

For peer review only - http://bmjopen.bmj.com/site/about/guidelines.xhtml

STROBE 2007 (v4) Statement—Checklist of items that should be included in reports of *cross-sectional studies*

Section/Topic	Item #	Recommendation	Reported on page #
**Title and abstract**	1	(*a*) Indicate the study's design with a commonly used term in the title or the abstract	1
		(*b*) Provide in the abstract an informative and balanced summary of what was done and what was found	2
**Introduction**			
Background/rationale	2	Explain the scientific background and rationale for the investigation being reported	4-5
Objectives	3	State specific objectives, including any prespecified hypotheses	6 (lines 1-2)
**Methods**			
Study design	4	Present key elements of study design early in the paper	6
Setting	5	Describe the setting, locations, and relevant dates, including periods of recruitment, exposure, follow-up, and data collection	6
Participants	6	(*a*) Give the eligibility criteria, and the sources and methods of selection of participants	6
Variables	7	Clearly define all outcomes, exposures, predictors, potential confounders, and effect modifiers. Give diagnostic criteria, if applicable	7-10
Data sources/ measurement	8*	For each variable of interest, give sources of data and details of methods of assessment (measurement). Describe comparability of assessment methods if there is more than one group	7-10
Bias	9	Describe any efforts to address potential sources of bias	6 (lines 15-20), 8(lines 6-12)
Study size	10	Explain how the study size was arrived at	6 (though not explained for our study)
Quantitative variables	11	Explain how quantitative variables were handled in the analyses. If applicable, describe which groupings were chosen and why	7-10
Statistical methods	12	(*a*) Describe all statistical methods, including those used to control for confounding	11
		(*b*) Describe any methods used to examine subgroups and interactions	Not done
		(*c*) Explain how missing data were addressed	11

For peer review only

		(*d*) If applicable, describe analytical methods taking account of sampling strategy	Not applicable
		(*e*) Describe any sensitivity analyses	Not done due to small missing data
**Results**			
Participants	13*	(a) Report numbers of individuals at each stage of study—eg numbers potentially eligible, examined for eligibility, confirmed eligible, included in the study, completing follow-up, and analysed	12
		(b) Give reasons for non-participation at each stage	12
		(c) Consider use of a flow diagram	Not necessary
Descriptive data	14*	(a) Give characteristics of study participants (eg demographic, clinical, social) and information on exposures and potential confounders	12
		(b) Indicate number of participants with missing data for each variable of interest	13, footnote Table 1; 14, footnote Table 2
Outcome data	15*	Report numbers of outcome events or summary measures	12
Main results	16	(*a*) Give unadjusted estimates and, if applicable, confounder-adjusted estimates and their precision (eg, 95% confidence interval). Make clear which confounders were adjusted for and why they were included	15
		(*b*) Report category boundaries when continuous variables were categorized	Not applicable
		(*c*) If relevant, consider translating estimates of relative risk into absolute risk for a meaningful time period	Not applicable
Other analyses	17	Report other analyses done—eg analyses of subgroups and interactions, and sensitivity analyses	Not done
**Discussion**			
Key results	18	Summarise key results with reference to study objectives	16
Limitations	19	Discuss limitations of the study, taking into account sources of potential bias or imprecision. Discuss both direction and magnitude of any potential bias	19-20
Interpretation	20	Give a cautious overall interpretation of results considering objectives, limitations, multiplicity of analyses, results from similar studies, and other relevant evidence	16-19
Generalisability	21	Discuss the generalisability (external validity) of the study results	19 (lines 16-18),
**Other information**			
Funding	22	Give the source of funding and the role of the funders for the present study and, if applicable, for the original study on which the present article is based	21

*Give information separately for cases and controls in case-control studies and, if applicable, for exposed and unexposed groups in cohort and cross-sectional studies.

For peer review only

Note: An Explanation and Elaboration article discusses each checklist item and gives methodological background and published examples of transparent reporting. The STROBE checklist is best used in conjunction with this article (freely available on the Web sites of PLoS Medicine at http://www.plosmedicine.org/, Annals of Internal Medicine at http://www.annals.org/, and Epidemiology at http://www.epidem.com/). Information on the STROBE Initiative is available at www.strobe-statement.org.

For peer review only
